# Structural basis for the dissociation of α-synuclein fibrils triggered by pressure perturbation of the hydrophobic core

**DOI:** 10.1038/srep37990

**Published:** 2016-11-30

**Authors:** Guilherme A. P. de Oliveira, Mayra de A. Marques, Carolina Cruzeiro-Silva, Yraima Cordeiro, Caroline Schuabb, Adolfo H. Moraes, Roland Winter, Hartmut Oschkinat, Debora Foguel, Mônica S. Freitas, Jerson L. Silva

**Affiliations:** 1 Programa de Biologia Estrutural, Instituto de Bioquímica Médica Leopoldo de Meis, Instituto Nacional de Biologia Estrutural e Bioimagem, Centro Nacional de Ressonância Magnética Nuclear Jiri Jonas, Universidade Federal do Rio de Janeiro, Rio de Janeiro, Brazil; 2 Faculdade de Farmácia, Universidade Federal do Rio de Janeiro, Rio de Janeiro, Brazil; 3 Department of Chemistry and Chemical Biology, Physical Chemistry, Technische Universität Dortmund, Dortmund, Germany; 4 Departamento de Química, Instituto de Ciências Exatas, Universidade Federal de Minas Gerais, Brazil; 5 Department of Structural Biology, Leibniz Institute für Molekulare Pharmakologie, Berlin, Germany

## Abstract

Parkinson’s disease is a neurological disease in which aggregated forms of the α-synuclein (α-syn) protein are found. We used high hydrostatic pressure (HHP) coupled with NMR spectroscopy to study the dissociation of α-syn fibril into monomers and evaluate their structural and dynamic properties. Different dynamic properties in the non-amyloid-β component (NAC), which constitutes the Greek-key hydrophobic core, and in the acidic C-terminal region of the protein were identified by HHP NMR spectroscopy. In addition, solid-state NMR revealed subtle differences in the HHP-disturbed fibril core, providing clues to how these species contribute to seeding α-syn aggregation. These findings show how pressure can populate so far undetected α-syn species, and they lay out a roadmap for fibril dissociation via pathways not previously observed using other approaches. Pressure perturbs the cavity-prone hydrophobic core of the fibrils by pushing water inward, thereby inducing the dissociation into monomers. Our study offers the molecular details of how hydrophobic interaction and the formation of water-excluded cavities jointly contribute to the assembly and stabilization of the fibrils. Understanding the molecular forces behind the formation of pathogenic fibrils uncovered by pressure perturbation will aid in the development of new therapeutics against Parkinson’s disease.

More than a century has passed since F.H. Lewy (1912) described the intracellular inclusion bodies of PD, but only in the 1990 s was the aggregated form of the α-synuclein (α-syn) protein shown to be part of the Lewy bodies (LBs) and neurites^1^. The decreased levels of dopamine in the substantia nigra pars compacta result in the characteristic motor symptoms of this neurodegenerative disorder. α-Syn aggregation is not linked exclusively to the most common form of PD but also to dementia with Lewy bodies, pure autonomic failure, multiple system atrophy (MSA), and autosomal dominant PD, in which missense mutations (A30P, A53T and E46K) are present^2^,^3^. In addition, α-syn gene multiplication and single-nucleotide polymorphisms are associated with several neurological syndromes and increased PD risk, respectively^4^,^5^,^6^.

Despite its link to neurodegeneration^1^,^2^, the physiological role of α-syn has not been fully explored. The 140-residue protein is localized either in the soluble form or bound to membranes in presynaptic nerve terminals (PNTs)^7^, and participates in the regulatory pathways of synaptic vesicle release and trafficking, modification of neurotransmitter release, neuronal survival, and plasticity^8^,^9^,^10^. Biochemical and biophysical studies have shown its intrinsically disordered behavior^11^ not only *in vitro* but also in neuronal cells^12^, although a tetrameric form has been described in *E. coli* (recombinant form), neurons, and red blood cells^13^,^14^. The protein has three regions: an N-terminal amphipathic segment that binds to lipids and forms α-helices^15^,^16^ a non-amyloid-β component (NAC), and an acidic C-terminus. Recent investigations have shown that in addition to the N-terminal region, the NAC and C-terminus also participate in the membrane binding process^17^. The membrane-bound α-syn may also trigger protein aggregation and seeding of the cytosolic form, which might explain some of the features observed in PD^7^,^18^.

There is a link between α-syn oligomerization and fibrillation and the cytopathological and neuropathological features of PD brains, which in turn are linked to the clinical symptoms of PD. Current efforts to ameliorate the devastating symptoms include stabilization of the monomeric form and blockage of the toxic supramolecular oligomers and fibrils^19^. High hydrostatic pressure (HHP) has become a strong physicochemical strategy for understanding the assembly of supramolecular structures such as amyloids^20^. Pressure mainly exerts its effects by promoting water infiltration into water-excluded cavities in the folded and assembled states^21^,^22^. However, there have been few studies exploring the use of HHP to investigate the molecular mechanisms underlying fibril disassembly and the conversion to species that might act as seeds for disease propagation and transmission^23^,^24^. There is substantial evidence supporting that PD is a prion-like disorder^25^,^26^,^27^,^28^,^29^ but the species that contribute to its seeding behavior have yet to be defined. For example, it is unclear why α-syn derived from patients with MSA exhibits a prion-like transmission character, whereas α-syn from Parkinson’s patients does not^30^. The existence of packing defects in the α-syn fibril core presents a valuable model for exploring the enrichment of potential intermediate species for fibrillogenesis and might shed light on new mechanisms interfering with fibril formation. The recently published structure of a toxic α-syn fibril reveals a structural topology consisting of a Greek-key motif with multiple β-strands intercalated with steric zippers, generating a compact, hydrophobic core^31^ that may explain why these fibrils are sensitive to pressure^23^,^24^. The folding of proteins into globular or fibrillar states is based on the formation of water-excluded cavities that can be perturbed by pressure^20^,^32^,^33^,^34^.

In this work, we identified the mechanism through which pressure triggers α-syn amyloid fibrils to be dissociated into the monomeric form. We provide molecular evidence of how hydrophobic interaction and the formation of water-excluded cavities jointly contribute to the assembly and stabilization of the fibrils. The high-pressure NMR and osmolyte data reveal that pressure pushes water into the hydrophobic core of the fibril, releasing monomers with altered dynamics. We investigated the structural and dynamic properties of these monomers dissociated from HHP-disturbed fibrils and the remaining fibrillar species at the atomic level, and examined how these species might seed amyloid fibril formation.

## Results

### Initial characterization of α-syn monomer and fibrils for HHP-NMR analysis

**Figure 1:**
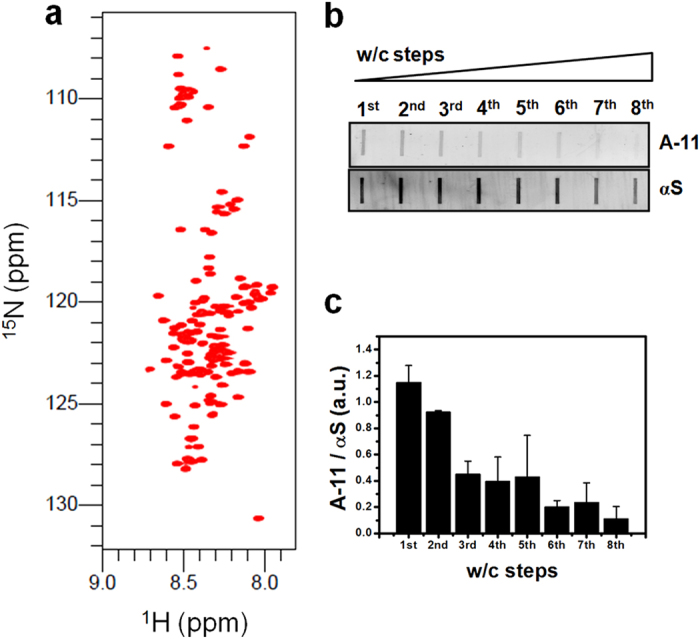
Initial characterization of α-syn monomers and fibrils for the HHP-NMR studies. (**a**) ^1^H-^15^N HSQC spectra of α-syn (αS) monomers. (**b**) The washing/centrifugation (w/c) steps were indirectly probed by dot-blots to exclude any remaining oligomeric species formed during the fibrillation reaction. We used eight w/c steps to decrease the amount of oligomers remaining in the fibril samples, as determined by the A-11 antibody signal. (**c**) Quantification of dot-blots was performed by densitometric analysis using the ImageJ software. The results are shown as the avg ± s.d. of three independent experiments.

First, the production of α-syn monomers and fibrils for HHP-NMR was characterized by a combination of spectroscopic and biochemical approaches. Although SDS-PAGE of monomers showed a size of ~17 kD, size exclusion chromatography (SEC) revealed a high-purity α-syn in the retention volume (V_*r*_) close to the value observed for the 44 kD chicken ovalbumin molecular standard (Supplementary Fig. 1a and inset), confirming the same behavior as previously observed by SEC^35^. Next, NMR assignment of the α-syn monomer was completed (Fig. 1a and Supplementary Fig. 2). The amyloid nature of the fibrils was observed through the increased quantum yield of Thioflavin T (ThT), x-ray scattering, and bis-ANS solvent-exposed hydrophobic surface area (Supplementary Fig. 1b–d). In addition, the increased β-sheet secondary structure of fibrils is shown by circular dichroism as a negative peak at approximately 220 nm, in contrast to the random conformation of monomers with a negative peak at approximately 200 nm (Supplementary Fig. 1e).

To rule out the contribution of residual monomeric or oligomeric α-syn species to the observed effect of increased pressure on fibrils, we excluded any remaining species from the fibril samples subjected to HHP-NMR studies (Fig. 1b,c). Fibrils were confirmed by transmission electron microscopy (Supplementary Fig. 1f) and subjected to a washing/centrifugation (w/c) protocol (see Methods) to remove undesired species without significantly affecting the α-syn fibrils in solution. Only at the eighth wash was there an apparent decrease in fibril concentration (Supplementary Fig. 3a). Following each washing, the oligomeric species washed away from fibril samples were indirectly assessed by dot-blots for an oligomer-sensitive antibody^35^,^36^. After eight washings, a very weak signal was observed (Fig. 1b,c). The exclusion of monomers after the w/c steps was confirmed by the ^1^H–^15^N HSQC spectra of fibrils at 1 bar, in which no signals were observed at the same threshold level as the monomer (Supplementary Fig. 3b). Thus, we established an initial condition for sample preparation in which α-syn fibrils are the predominant species sensing HHP effects when observed by NMR.

### Species released from α-syn fibrils upon HHP

**Figure 2:**
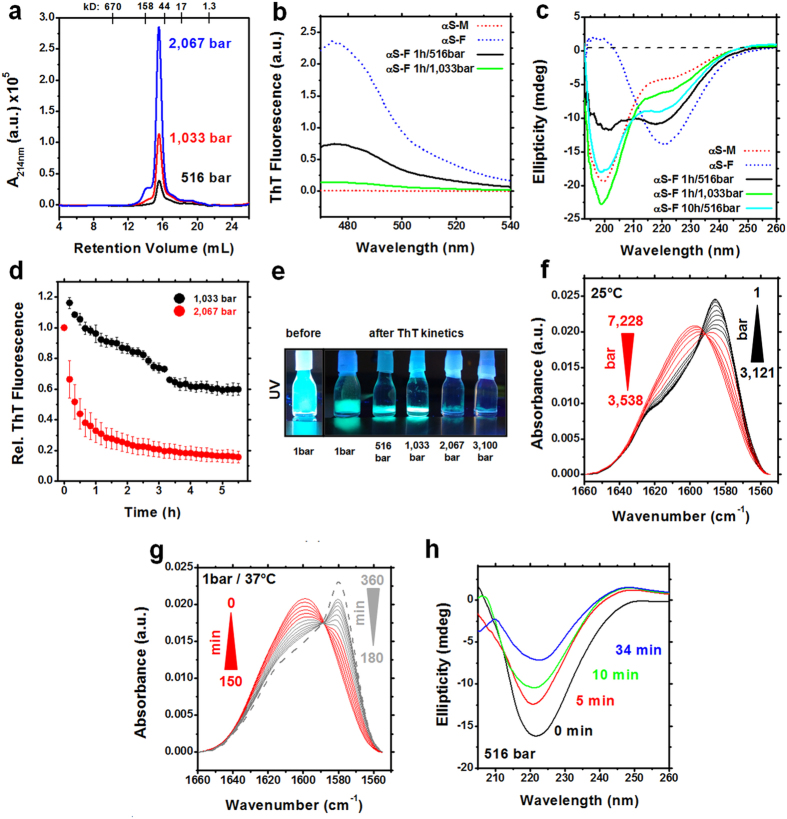
Species released from α-syn fibrils under HHP. (**a**–**c**) HHP-disturbed fibrils at 1 bar. (**a**) SEC in Superdex 200 10/300 of fibrils after 1 h of treatment at 516, 1,033, and 2,067 bar. Absorbance at 214 nm of monomeric α-syn peak increased as HHP increments were applied to fibrils. (**b**) ThT fluorescence signal of fibrils (αS-F) decreased similarly to the signals of monomers (αS-M) after 1 h at 1,033 bar. (**c**) The ellipticity of fibrils subjected to HHP shifted from β-shift secondary structure (negative peak at 220 nm) to a random conformation (negative peak at 200 nm), which is typical of α-syn monomers. (**d**) Disassembly kinetics of sedimented fibrils at 1,033 (black) and 2,067 (red), as monitored by the loss of ThT fluorescence. Sedimented fibrils were subjected to these pressure values for the whole kinetics immediately after ThT signal stabilization at 1 bar, which was achieved after 3 h. Measurements are shown as avg. ± s.e.m. (*n* = 3, independent protein batches). (**e**) Visual inspection of ThT signal under UV light of non-sedimented fibrils (before) and sedimented fibrils (after 3 h at 1 bar) and after ca. 6 h subjected to 516, 1,033, 2,067, and 3,100 bar of pressure increments. (**f**) The absorbance of the amide I′ band as a function of pressure increments of^15^N/^13^C α-syn fibrils by HHP-FTIR. The pressure ranges from 1 to 3,121 bar and from 3,538 to 7,228 bar are shown in black and red, respectively, to highlight the shifted peaks. (**g**) Amide I′ band recovery at 37 °C was monitored by FTIR spectroscopy every 30 min after pressure release for 360 min. Red and gray lines represent the shift recovery over time. The dashed gray line representsthe amide I’ band after 12 h. (**h**) Ellipticity response over time to 516 bar, as measured by HHP-CD.

We used HHP to obtain insights on the species released from α-syn fibrils. Washed fibrils subjected to increasing HHP revealed a major population of monomeric species as evaluated by size exclusion, but no oligomeric species were detected (Fig. 2a). Furthermore, the ThT fluorescence of fibrils after 516 and 1,033 bar treatment revealed an abrupt signal decrease and, by circular dichroism, a major shift from the β-sheet signal to the random conformation expected for monomeric α-syn (Fig. 2b,c).

To better understand the disassembly of fibrils at 1,033 and 2,067 bar, we recorded the ThT kinetics at these pressures after ThT signal stabilization at 1 bar (i.e., sedimented fibrils; Fig. 2d,e and Supplementary Fig. 3c,d). The data show that fibril disassembly is pressure dependent and a complex process, especially at 1,033 bar, where two decaying profiles are observed throughout the kinetics (Fig. 2d). To determine further details of the effects of pressure on the fibril’s secondary structure, we measured the real-time pressure dependence by Fourier transform infrared (FTIR) spectroscopy (Fig. 2f,g) and circular dichroism (Fig. 2h). HHP-CD measurements at 516 bar over time revealed a continuous decrease of the β-sheet signal at 220 nm, consistent with the process of fibril dissociation. ^15^N/^13^C fibrils were used to follow amide I changes by HHP-FTIR. We observed a systematic change in the amide I band with increasing pressure (Fig. 2f). The deconvolution of FTIR spectra at 1 bar (major band at 1,588 cm^−1^, corresponding to 1,630 cm^−1^ for non-labeled samples) fitted the absorption interval of β-strands. At 7,228 bar, two major bands at 1,602 and 1,588 cm^−1^ (1,651 and 1,640 cm^−1^ for non-labeled) were predominant, corresponding to irregular structures (Supplementary Fig. 4a–c). Finally, we showed the ability of α-syn monomers dissociated from fibrils to recover the β-strand absorption band, which was consistent with the process of *de novo* aggregation (Fig. 2g). Spectral analysis and processing established a decrease in the β-sheet content and a slight increase of α-helix, random coils and turns/bends, consistent to the process of fibril dissociation to monomers. Altogether, our HHP-FTIR and -CD data provided real-time measurements of pressure effects on α-syn fibrils, suggesting that pressure-induced dissociation probably cause formation of disordered monomers.

**Figure 3:**
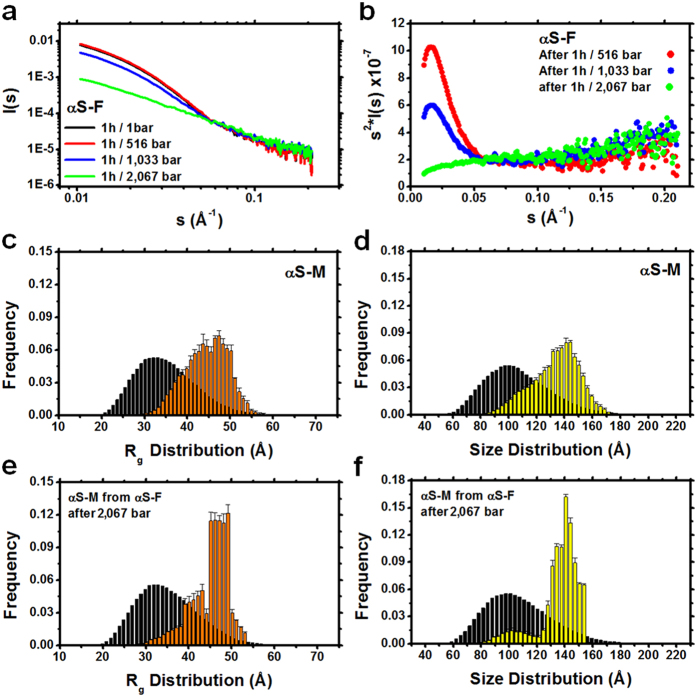
Small-angle X-ray scattering profile of α-syn species released from fibrils. (**a**) Scattering intensity *I*(*s*) as a function of the scattering vector *s* of α-syn fibrils (αS-F) at 1 and after 516, 1,033, and 2,067 bar for 1 h. Scattering was measured at atmospheric pressure. (**b**) Kratky plots showing the shift of the folding pattern of fibrils to the flexible and unfolded behavior of species dissociated from fibril safter HHP treatment. (**c**–**f**) Frequency of radius of gyration, *R*_g_ (orange), and size distribution (yellow) of initial monomers (**c**,**d**) and species released from fibrils after 1 h of 2,067 bar treatment (**e**,**f**), as evaluated by the ensemble optimization method. Black bars represent the distribution of the pool of conformers (a collection of 10,000 random conformers) used to define the conformational space.

Further evaluation of small-angle x-ray scattering profiles from fibrils after HHP increments has shown a lower scattering intensity as a function of the scattering vector *s* (Fig. 3a) and Kratky plots after 1 h/2,067 bar with increased values of *s*^2^*I*(*s*) vs. *s* (Kratky-plot), which is consistent with species adopting increased flexibility due to fibril disassembly (Fig. 3b). The scattering of monomers at different concentrations (Supplementary Fig. 5a–c) revealed very similar scattering patterns and Kratky plots as compared to the results obtained from fibrils after HHP treatment at 2,067 bar. To observe the frequency distribution of the species dissociated from fibrils, we performed an ensemble optimization method (EOM) on the scattering data of monomers not subjected to pressure and of species obtained from HHP-disturbed fibrils (Fig. 3c–f and Supplementary Fig. 5d,e). We observed that the species dissociated from fibrils presented a narrower population enriched in conformers of approximately 45–50 Å in radius of gyration, *R*_g_, and 140–150 Å in size distribution (Fig. 3e,f), revealing that the ensemble of species released from fibrils are not the same as the species in the absence of HHP treatment and are not uniformly distributed throughout the conformational space.

### α-Syn fibril dissociation into monomers, as monitored by HHP-NMR

**Figure 4:**
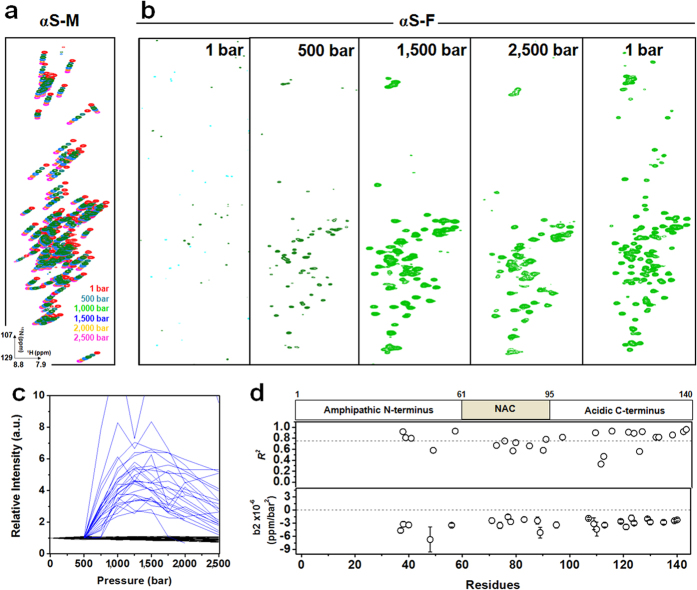
α-Syn fibril dissociation into monomers, as monitored by HHP-NMR. (**a**) Superposition of ^1^H-^15^N HSQC spectra of monomeric α-syn (αS-M) at different pressure increments ranging from 1 to 2,500 bar. The systematic chemical shifts of the^1^H-^15^N correlations with increasing pressure are the result of the compressibility effects pressure imposes on the solvent hydrogen bonds to the protein backbone. (**b**) HHP titration of α-syn fibrils (αS-F) using pressure increments higher than 500 bar revealed ^1^H-^15^N correlations typical of the monomeric protein. (**c**) Line-broadening analysis of the response to increasing pressure for monomeric α-syn (black lines) and for monomeric species released from the fibril (blue lines). Each line corresponds to one ^1^H-^15^N correlation in which we were able to follow the line-broadening behavior as a function of increasing pressure. (**d**) The *r*-square (*R*^*2*^) values and the second-order coefficients (*b*2) as a function of residues obtained after fitting the bell-shaped behavior of the α-syn species released from fibrils to a second-order polynomial equation.

We next asked whether we could monitor the HHP-induced fibril disassembly at the atomic level. To identify the species dissociated from the fibril, we set up HHP titrations and monitored the changes in the ^1^H–^15^N HSQC spectra under pressure. Washed fibrils were subjected to increasing pressures, and at each pressure increment, the HSQC peaks obtained from dissociated species were compared to the HSQC peaks obtained for initial monomers at the same pressure (Fig. 4a and Supplementary Fig. 6). The superposition of spectra obtained from species dissociated from fibrils and initial monomers not incorporated into the fibrils at different pressure values were particularly important to exclude the compressibility effect on monomers and to allow the tracking of the α-syn species released from the fibrils by applying pressure to the fibril core (Fig. 4b and Supplementary Fig. 6). These data provide atomic-level information on the monomeric species being released from fibrils. First, systematic chemical shifts of the initial monomers not incorporated into the fibril core occurred due to the compressibility effects of increasing pressure on the protein backbone and were monitored by chemical shift perturbation (CSP) analysis at 500, 1,000, and 2,500 bar and compared to the spectra at 1 bar (Supplementary Fig. 7a). Several cross peaks at residues located in the C-terminal region of the amphipathic N-terminus (Y39, S42, T44, T54, T59) as well as those in the NAC region (T64, T75, T81, T92, and V95) exhibited CSP values higher than the avg. ± s.d. among the evaluated residues (Supplementary Fig. 7b), revealing the most labile segments in the α-syn monomers upon compression.

Pressure effects on fibril disassembly were observed by ^1^H–^15^N HSQC, starting at 500 bar and monitored up to 2,500 bar and after the release of the pressure (Fig. 4b). Signals due to species dissociated from fibrils were consistent with α-syn monomers and assigned at a specific pressure value, and compared to the initial monomer assignment at the same pressure. Among the 100 correlations assigned for initial monomers at 1 bar, we were able to assign for the dissociated species from fibrils, 40 correlations at 500 bar, 55 at 1,000 bar, 59 at 1,500 bar, 34 at 2,500 bar, and 93 upon pressure release (Fig. 4b). The intensities from each peak in the ^1^H–^15^N HSQC spectra relative to the values at 1 bar for monomers that were not incorporated into the fibrils (Fig. 4c, black lines) and to the values of 500 bar for dissociated species released from the α-syn fibrils (Fig. 4c, blue lines) were plotted as a function of pressure increase. This plot revealed a different behavior of the monomeric sample compared to monomeric species released from fibrils upon HHP treatment. While there were no changes in the relative intensities (line-broadening effects) for the initial monomers, the monomers released from fibrils exhibited a bell-shaped behavior (26 studied correlations) and were fitted to a second order equation that reflects conformational exchange motions^37^,^38^. The second-order coefficients (b2) of 7 correlations in the NAC region and 14 correlations in the acidic C-terminus were monitored as a function of residue number and revealed significant negative values consistent with conformational exchange motions in these regions (Fig. 4d). These results suggest that the monomeric species released from α-syn fibrils are structure-modified monomers (SMMs) presenting dynamic behavior in the conformational exchange regime. Interestingly, part of the mapped region belongs to the hydrophobic core of the fibril that is stabilized by water-excluded cavities and a salt bridge, with steric zippers forming the dry interface^31^. Water-excluded cavities and salt bridges are highly sensitive to pressure^20^,^32^.

**Figure 5:**
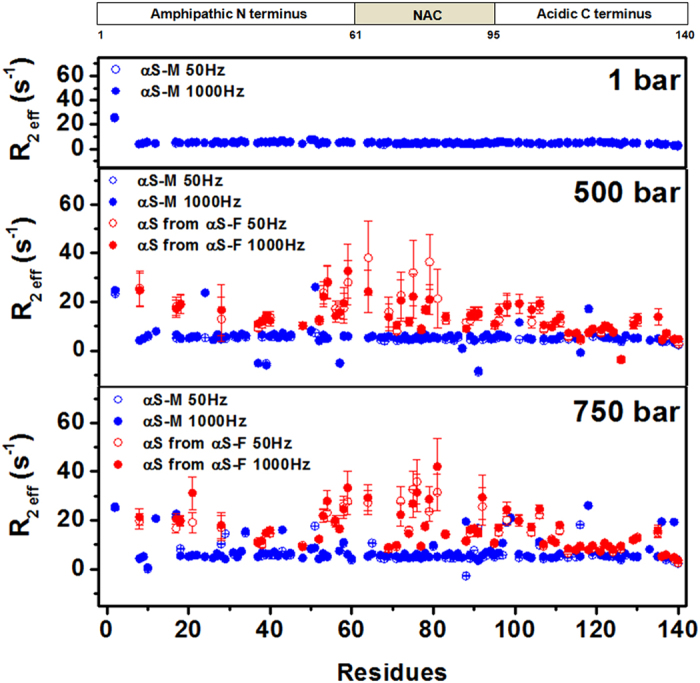
Conformational dynamics of monomeric species released from HHP-disturbed fibrils. Relaxation rates (*R*_2 eff_) obtained from the intensities of resonance in ^1^H-^15^N correlation spectra acquired with 50 (open symbols) and 1000 Hz (filled symbols) for initial monomers (αS-M, blue) at 1, 500 and 750 bar and from species released from fibrils (αS from αS-F, red) at the same pressure values. All experiments with monomeric α-syn and fibrillar samples were taken at the specified pressure after 20 minutes of equilibration. Experiments were run once and error bars represent deviations from noise peaks.

**Figure 6:**
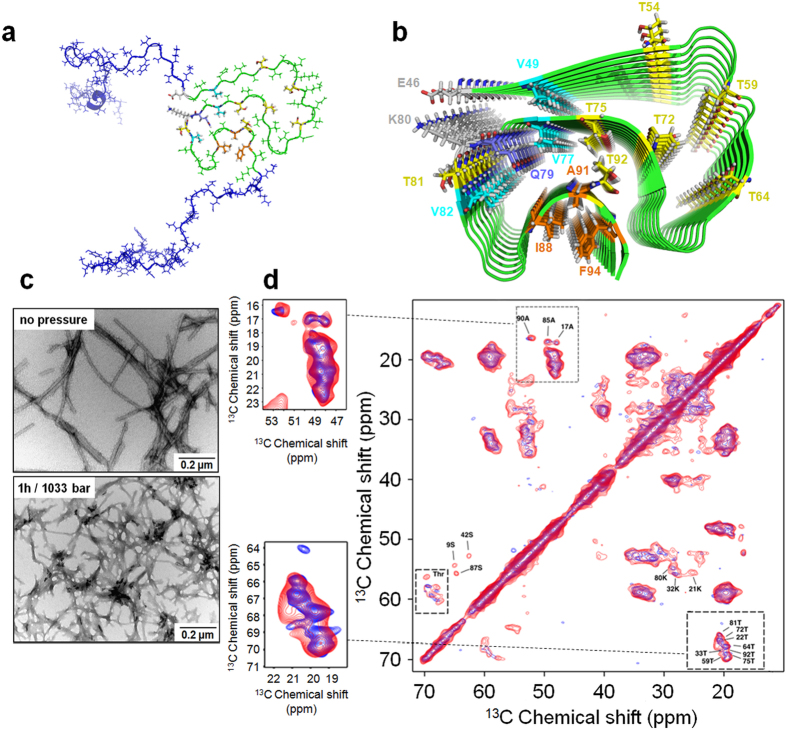
HHP effects on fibril structure. (**a**) Solid-state NMR structure of full-length α-syn monomer (PDB: 2n0a) highlighting the N- and C-terminus (blue) and the Greek-key arrangement of the core (green). (**b**) Solid-state NMR structure of the α-syn fibril core (residues 46–96, PDB: 2n0a) showing the key elements for fibril stability: the salt-bridge between E46-K80 (gray), steric zippers involving V49, V77 and V82 (cyan), the glutamine ladder Q79 (purple) and the hydrophobic packing involving I88, A91 and F94 (orange). Threonine positions (yellow) are highlighted to show the effects of HHP on the α-syn fibril core. (**c**) Negatively stained transmission electron microscopy of fibrils before and after 1 h treatment at 1,033 bar. (**d**) ^13^C–^13^C correlation spectrum of the α-syn fibril core acquired in a static magnetic field of 600 MHz before (blue) and after (red) 1 h at 1,033 bar. Dashed lines show the changes.

For an in-depth exploration of the dynamic behavior of the SMMs released from α-syn fibrils, we evaluated the pressure dependence of these species and compared them with free monomers by performing ^15^N Carr-Purcell-Meiboom-Gill relaxation dispersion (CPMG-RD) experiments (Fig. 5). Of particular interest is the non-amyloid-β component (NAC) and the first half of the acidic C-terminal region, which showed the highest *R*_2_ values. This particular region forms the hydrophobic core of the fibril, as recently revealed by ssNMR^31^. As depicted in the structure of the fibril, this region has key elements including a salt bridge and dried hydrophobic bulks that are highly susceptible to pressure (Fig. 6a,b). Our HHP CPMG-RD analysis shows that SMMs released from α-syn fibrils present a different dynamic behavior from the initial monomers, and a complex regime of motions occurs upon dissociation (Fig. 5). Hence, our data strongly support the existence of so far undetected invisible monomeric conformers released from fibrils upon HHP challenge that present a different dynamic behavior from monomers that were never incorporated into the fibril core.

### HHP effects on fibril structure

To evaluate if remaining fibrils adopt a different structure after mild pressure challenges we designed electron microscopy and solid-state NMR experiments. We found that they do indeed have a different morphology, as detected by negatively stained transmission electron microscopy images (Fig. 6c). After pressure treatment, fibrils seems to get thicker and form clusters, probably due to pressurization effects on fibril dissociation. Of note, pre-dissociated and pre-denatured states have also been found in small proteins^20^,^39^.

To test whether the core of the α-syn fibrils was affected (before disassembly), a solid-state NMR approach was applied to assess the structural differences between fibrils before and after HHP treatment at 1,033 bar. Because α-syn includes ten threonine residues, and nine belong to the fibrillar core^31^,^40^,^41^, the effects of HHP on the fibril structure were probed by the difference in threonine chemical shifts. Based on the ^13^C–^13^C correlation spectra, it was possible to verify a slight difference in the threonine chemical shifts before and after HHP treatment, suggesting small changes in the remaining fibrillar population (Fig. 6d). To confirm our results, a second batch of fibrils was prepared, corroborating the result obtained for the first batch (Supplementary Fig. 8). For a more in-depth evaluation of fibril changes under pressure, it would be important to observe the linewidth of threonine peaks. In such a case, a less polymorphic sample should be used to provide narrow lines.

### Seeding mechanism of HHP-disturbed fibrils

**Figure 7:**
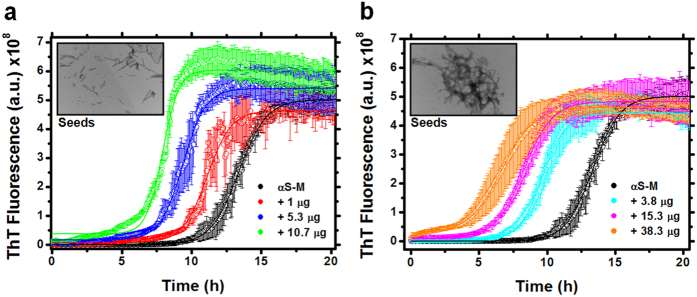
Seeding of remaining fibrils after HHP treatment. Aggregation kinetics of α-syn monomers (αS-M) in the absence of seeds (black spheres) or in the presence of increasing micrograms of (**a**) sonicated fibrils and (**b**) structural modified monomers (SMMs) + remaining fibrils formed after 1 h incubation of fibrils at 1,033 bar. Measurements of the ThT fluorescence are shown as avg. ± s.d. of three independent experiments. Insets show negatively stained electron microscopy images of seeds used for the kinetic experiments.

Following the identification and characterization of the main species released from fibrils and the remaining fibrils after 1,033 bar treatment, we examined the effect of these species on seeding the aggregation of α-syn monomers. By recording the fluorescence intensity of ThT as a function of time, we followed the aggregation of α-syn when it was incubated with different seeds (Fig. 7 and Supplementary Figs 9a–d and 10a–h). Because the conditions used to produce α-syn seeds affect the final concentration of fibrils, we first developed a reliable method to determine fibril concentrations after seed production (Supplementary Fig. 9a). As expected, the sonication protocol did not affect the fibril concentration compared to non-treated fibrils. In contrast, seeds produced by HHP at 1,033 and 2,067 bar resulted in an average reduction of ca. 34% and 85% compared to non-treated fibrils, respectively, as determined by A_280 nm_. Our data show that the remaining species (Rm-F) formed at 1,033 bar (meaning SMMs + Rm-F) were able to seed α-syn aggregation, but less efficiently than seeds formed after fibril sonication, as revealed by the t_1/2_values (Fig. 7a,b and Supplementary Fig. 9b). The species remaining after 2,067 bar treatment (SMMs + Rm-F) and non-treated fibrils have a modest effect on seeding only compared to the species formed after the 1,033 bar treatment (Supplementary Fig. 9b–d). Of note, we did not observe changes in the slope values of transitions when comparing increasing amounts of seeds from remaining fibrils after 1,033 bar treatment, in contrast to the values obtained for sonicated fibrils (Supplementary Fig. 10i–l). Altogether, we show that different seeding mechanisms may contribute to the balance between the primary nucleation and elongation phases.

### The mechanism of fibril disassembly triggered by pressure needs water

**Figure 8:**
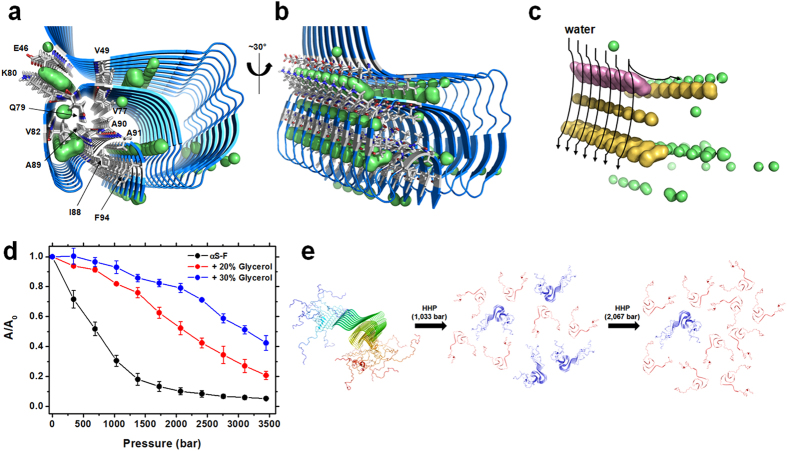
Mechanism of dissociation of α-syn fibrils by pressure. (**a**,**b**) The cavity-prone hydrophobic core of α-syn fibrils (PDB: 2n0a). Non-exposed cavities along the fibril axis and residues located in the vicinity of these cavities are highlighted as green surfaces and gray sticks, respectively. (**c**) Hydration of the hydrophobic core triggered by pressure. The cavity that may sense hydration first (purple) is the one formed by the side chains of the E46-K80 pair, followed by the ones located inward toward the cavity-prone hydrophobic bulk (orange). The presence of other small cavities (green) may ultimately disrupt the fibril core. (**d**) Light scattering (measured as the area under the light scattering curve A and normalized by the value at 1 bar A_0_) of α-syn fibrils in the absence or in the presence of 20 and 30% of glycerol. Measurements are shown as avg. ± s.e.m. (*n* = 3, independent protein preparations). (**e**) Schematic representation of pressure effects on α-syn fibrils, showing the major findings obtained by challenging α-syn fibrils with pressure. Structurally modified monomers (SMMs) are shown in red and remaining fibrils in blue.

To better understand the mechanism under which pressure leads to fibril disassembly we used the recent solid-state NMR structure of the α-syn fibril and a Monte Carlo algorithm to explore the existence of non-exposed cavities (Fig. 8a–c). Interestingly, the side chains of the intermolecular salt-bridge pair E46-K80 form a non-exposed cavity along the perpendicular axis of the fibril, as clearly visualized in Fig. 8. Furthermore, the cavity-prone hydrophobic core formed by the side chains of Q79, V82, and A89 accommodates two additional cavities immediately inward from the Greek-key topology that might be facilitated by the presence of several small residues in the turn segments (Fig. 8a–c). The locking mechanism of the α-syn fibril core consisting of the charges of the E46-K80 pair and the corresponding non-exposed cavity formed by these residues would likely make it a vulnerable site into which water molecules get pushed and ultimately hydrate the cavity-prone hydrophobic core (Fig. 8). To assess the relevance of water on fibril disassembly we have used glycerol. Because this osmolyte preferentially excludes water molecules from around the fibril assembly and also from the immediate vicinity of the dissociated species, we expected the pressure-induced release of monomers to be progressively less effective as the concentration of glycerol increased. The measurement of light scattering as a function of pressure increments revealed that fibrils are less affected as increasing glycerol concentrations is used, confirming the important role of water molecules on fibril disassembly (Fig. 8d). The three aligned non-exposed cavities may explain the peeling of monomeric species from the fibril at mild pressure due to water penetration (Figs 6c,d and 8a–d).

## Discussion

We demonstrate that the major ensemble of species released from HHP-disturbed fibrils are monomers that present a different dynamic behavior at the NAC and acidic C-terminal regions. Moreover, the remaining fibrils are slightly different at the level of the core region. Interestingly, while we do not detect appreciable amounts of oligomers upon dissociation of α-syn, we do detect slightly different fibrils as pre-dissociated species. The presence of key elements including a salt-bridge and water-excluded cavities along the perpendicular axis of the dried hydrophobic core explains its susceptibility to pressure (Fig. 8a–c).

It has been shown that the aggressive variants A30P and A53T are more susceptible to pressure than the wild-type as monitored by light scattering measurements^23^. This finding reveals the existence of different packing defects and hydrophobic pockets in the fibril core^23^, rendering α-syn fibrils a valuable model for investigating HHP-induced intermediates and unveiling fibrillation pathways. Furthermore, it has been shown that HHP strongly reduces the toxicity levels of A30P protofibrils in mesencephalic and cortical neurons by breaking them into smaller aggregates^24^. Finally, a recent study has shown that in neuronal cells challenged by HHP, α-syn loses contact with its PLCβ-1 partner, thereby triggering α-syn protein aggregation inside the cell^42^. These HHP studies on α-syn provide a wide range of HHP applications for improving our understanding of aggregation pathways, not only mechanistically but also functionally. Nevertheless, the molecular mechanism by which HHP triggers the dissociation of α-syn fibrils has never been addressed at the atomic level, limiting current interpretations to the field of bioimaging and biochemical approaches. Here, we explored the mechanism by which pressure dissociates α-syn fibrils, and add new information regarding the presence of so far invisible and dynamically modified monomeric species released from fibrils (Figs 2 and 4) and the remaining slightly modified fibrils (Fig. 6). Indeed, the hydration of exposed nonpolar residues, the presence of non-exposed cavities, and the electrostriction of exposed charges are key elements in explaining protein unfolding triggered by pressure^20^,^32^,^33^,^34^. The recent ssNMR structure of the Greek-key α-syn fibril provided the framework for understanding fibril stability^31^ and insights into how pressure would affect its core (Fig. 8e).

Although appreciable studies have shown the disaggregation of different fibrils triggered by osmotic and chemical agents^43^,^44^ the challenges of identifying and characterizing these transient and heterogeneous intermediate species remain notoriously difficult. Using single-molecule strategies and near-physiological conditions, α-syn fibrils were shown to disaggregate into monomers as well as oligomeric species, the latter dissociating into monomers after longer incubation times^45^. Here, the combination of pressure-based approaches not only provided insights into the mechanism of α-syn fibril disassembly but also yielded structural information on the dynamic properties of these intermediate monomeric species and the remaining fibrils formed after compression.

Several studies support that PD exhibits prion-like behavior^25^,^26^,^27^,^28^,^29^,^30^ but there is still no clear evidence regarding what species contribute to the mechanism of seeding and cell-to-cell spreading. Both monomeric and aggregated forms of α-syn have been previously detected in the cerebrospinal fluid and plasma^46^ in humans and found to be secreted by rat primary cortical neurons^28^. In our study, we show that the fibril-to-monomer transition occurs upon HHP challenge without appreciable amounts of oligomers, and that the dynamic properties of these SMMs differ from those of the initial α-syn monomers.

Considering that cells take up all three species of α-syn (monomers, oligomers, and fibrils) from the extracellular space^28^ that α-syn monomers do not induce pathology^29^, and that fibrillar aggregation is not exclusively the result of primary nucleation processes but is also catalyzed by mature amyloid fibrils, an event known as secondary nucleation^47^,^48^, we asked whether these remaining fibrillar species produced by HHP may play a role in seeding α-syn aggregation. Although fibrillar seeds formed after pressure treatment seem to have similar seeding properties to non-treated fibrils, they exhibit different morphologies and slightly different core structures, providing an opportunity for new platform-based studies for combating PD.

The use of seeds accelerates fibrillar aggregation reactions through elongation and surface-catalyzed secondary nucleation and decreases the lag phase, as observed in our seeding experiments (Fig. 7). In contrast to what has been shown for the aggregation of Aβ42, in which the association of primary and secondary events occurs by the growth of oligomeric nuclei and fibrils^49^, for α-syn, the amyloid fibril growth occurs through monomer but not oligomer addition^50^. The dissociation mechanism triggered by pressure points to the reverse of this assembly reaction. At this point in the study, the lacking information is whether these SMMs populated by HHP are trapped species in the amyloidogenic pathway of α-syn or may participate in the *de novo* formation of higher-order oligomers and fibrils. By FTIR, we observed the recovery of the β-sheet signal of these monomers released from HHP-disturbed fibrils at physiological temperature (Fig. 2g).

Using preformed α-syn fibrils produced by sonication as seeds, we show that in contrast to the observations of these sonicated fibrils, in which secondary nucleation dominates the elongation rate (as revealed by the increasing slopes at the transition points, Fig. 7a and Supplementary Fig. 10i–l), the seeds of remaining fibrils populated by HHP and non-treated fibrils do not exert any influence on the elongation phase of aggregation (equal slopes, Fig. 7b and Supplementary Fig. 10i–l). Therefore, these remaining fibrils may participate in seeding through primary nucleation processes such as providing hydrophobic-prone surfaces for fibril growth, previously reported as a requirement for α-syn fibrillation^51^.

In conclusion, we provide biochemical and structural data on the mechanism of how pressure affects the cavity-prone hydrophobic core of α-syn fibrils and leads to fibril dissociation of dynamic monomeric species (Fig. 8e), which were heretofore not detected by conventional techniques. HHP should be considered as a potential tool for developing a new generation of target species and for drug screening studies based on the dynamic properties of intermediate structures uncovered. Intermediate species of amyloidogenic pathways have already been observed in the case of transthyretin (TTR), which is involved in senile systemic amyloidosis and familial amyloidotic polyneuropathy^52^. Future therapeutics focused on the blockage of *de novo* aggregation and seeding and the development of new biomarkers for early diagnosis may represent an effective strategy to combat PD.

## Methods

### Preparation of monomeric α-syn and fibrils

Recombinant monomers were produced based on the previous protocol^53^. For protein purification, cells were harvested from 0.8 L culture and resuspended in 30 mL of 20 mM Tris-Cl (pH 8.0) containing 5 mM EDTA and 1 mM phenylmethanesulfonyl fluoride, homogenized for 1 to 3 sec in an automated homogenizer (Novatecnica) and lysed by ultrasonication in a Vibra cell machine for 20-sec intervals over 30 min at 300 W (Sonics&Materials, Inc.). The crude extract was then subjected to osmotic shock by decreasing the pH to 3.5 using 1 M HCl and centrifuged at 16,000 *g* for 20 min at 4 °C. Next, the pH of the supernatant was neutralized (pH 7.5) using 1MNaOH followed by α-syn precipitation with 50% w/v (NH_4_)_2_SO_4_ at 10 °C. After centrifugation (16,000 *g* for 20 min, 4 °C), the pellet was resuspended in ca. 30–40 mL of 20 mM Tris-Cl (pH 8.0) containing 1 mM EDTA and dialyzed overnight (ca. 12–16 h) against 2 L of the same buffer followed by two changes with MilliQ water (2 L for each change, same duration). Different aliquots from 8–10 mg (ca. 3–5 mL) of the dialyzed protein were flash frozen in liquid nitrogen and lyophilized for 36–48 h in a Flexi Dry unit (FTS Systems, #FD-1-84A) coupled with a vacuum pump. Lyophilized protein batches were stored at −20 °C and used after an interval no longer than one month. For ^15^N- or ^15^N–^13^C-labelled samples, the protein was produced as described previously^54^ and purified as above.

For fibril preparation, one lyophilized aliquot (ca. 8–10 mg) was resuspended in 1 mL of 10 mM Tris-Cl (pH 7.4) 100 mM NaCl and centrifuged at 20,000 *g*, 10 min, 4 °C. The supernatant was then injected four times (250 μL per injection at a flow rate of 0.7 mLmin^−1^) into a Superdex 75 10/300 column previously equilibrated in the same buffer (Supplementary Fig. 1a) using a Ultra Fast Liquid Chromatograph (Shimadzu). At this step, SEC was chosen to obtain α-syn at higher purity (>99%) (Supplementary Fig. 1a, inset). The concentration of monomeric α-syn was estimated by absorbance at 280 nm (A_280 nm_) using a molar extinction coefficient of 5,960 M^−1^ cm^−1^. Fibrillation reactions were performed using at least two different protein batches to a final volume of 1 mL in 2 mL low-protein binding tubes (Eppendorf) using 140 μM of α-syn obtained immediately after SEC. Samples were incubated at 37 °C and with continuous shaking at 600 rpm using a Thermomixer comfort (Eppendorf) for 7–10 days.

### Washing protocol of α-syn fibrils

Fibrils were submitted to a washing/centrifugation (w/c) protocol to exclude remaining species (α-syn monomers and oligomers) from the fibril solutions. Fibrils were washed (only by swirling the solution) eight times with 1 mL of 10 mM Tris-Cl, 100 mM NaCl pH 7.4 using 15 mL conical tubes and a swing bucket rotor at low velocity, 3,000 *g*, 10 min, 4 °C. This velocity was chosen because no significant changes were observed in the dissociation rate of α-syn fibrils measured at each washing step based on A_280 nm_ of an aliquot of resuspended fibrils dissolved in 5 M guanidinium chloride (Supplementary Fig. 3a). The α-syn fibril concentration used in the experiments was estimated by measuring A_280 nm_ from the monomeric fraction released from the fibril after treatment with 5 M guanidinium chloride (i.e., the monomeric α-syn concentration present in the fibril), and used to estimate the concentrations of seeding conditions (Supplementary Fig. 9a).

### Size exclusion chromatography in ultra-fast liquid chromatography (UFLC)

Size exclusion chromatography (SEC) for the α-syn species formed after HHP treatment were performed using two different columns: a GPC-250 (Agilent) and a Superdex 200 10/300 (GE Life science). Equal amounts of washed fibrils (87 μM) were subjected to 1, 516, 1,033, and 2,067 bar for 1 h, followed by centrifugation at 20,000 *g*, 10 min, 4 °C and injection of the supernatants. All UFLC runs were performed in 10 mM Tris-Cl (pH 7.4), 100 mM NaCl at a flow rate of 0.7 mL min^−1^, and the absorbance was monitored at 214 nm. The elution volumes from each species were always compared with a molecular weight protein standard (Biorad, #151-1901). Both columns presented similar elution profiles with no detectable amounts of oligomeric species in the studied concentration range.

### Routine transmission electron microscopy (TEM)

TEM images were obtained after 7 days. Samples 3 μL in volume were applied for 3 min to previously discharged carbon film on 200 mesh cooper grids (EMS, #CF200-Cu), gently dried with filter paper and stained for 1 min with 5% uranyl acetate. Negatively stained samples were visualized on a Zeiss 902 microscope operated at 80 kV with different magnifications (30,000×, 50,000×, 80,000×).

### Dot-Blots (DBs)

DBs probed with anti-α-syn (Invitrogen, #AHB0261, batch 1031405c) and A-11 (Millipore EMD, #AB9234, batch 2387440) antibodies were used during the w/c steps of α-syn fibrils to evaluate the rate of oligomeric species washed away from fibril samples. The A-11 antibody has been reported to recognize oligomeric species of different amyloidogenic proteins, including α-syn^35^,^36^. DBs were performed by applying equal amounts of resuspended α-syn fibrils after each w/c step to a 0.45 μm Immobilon-FL low fluorescence PVDF membrane (Millipore, # IPFL00010) mounted on a 48-well Bio-Dot (slot format) microfiltration apparatus (Bio-Rad). Samples were vacuum-filtered and blocked overnight (ca. 12–16 h) at 4 °C under mild agitation with 5 mL of Odyssey blocking buffer - TBS (LI-COR, # 927-50000). Next, the membranes were probed separately at 4 °C overnight under mild agitation with anti-α-syn and A-11 primary antibodies at 1:1,000 dilution in the Odyssey blocking buffer. The membranes were then washed four times for 5 min with TBS containing 0.1% Tween-20 and finally incubated for 1 h at 4 °C with IRDye 800CW anti-mouse (LI-COR) for α-syn and IRDye 800CW anti-rabbit (LI-COR) for A-11 at 1:10,000 dilution. Then, the membranes were again washed four times for 5 min with TBS containing 0.1% Tween-20, and infrared fluorescence images were acquired using the Odyssey system (LI-COR) and quantified using the ImageJ software.

### Fluorescence spectroscopy (FS) and HHP-FS

The fluorescent probes used to measure the α-syn monomers and fibrils were Thioflavin T (ThT) and 4,4′-dianilino-1,1′binaphthyl-5,5′-disulfonic acid (bis-ANS). Measurements were performed at 25 °C on a ISSK2 spectrofluorometer (ISS, Inc.) equipped with a high-pressure cell (ISS, Inc.). Bis-ANS and ThT binding to α-syn fibrils were monitored by exciting samples at 360 and 450 nm, respectively, and recording the emission spectra at 400–600 nm (for bis-ANS) and 460–540 nm (for ThT) at 1 bar. For ThT binding, samples were incubated for 1 h at 516 and 1,033 bar before spectrum acquisition at atmospheric pressure. Fibrils at ca. 45–60 μM and monomers at 55 μM were incubated with 10 μM ThT prepared in water or 25 μM bis-ANS for 10 min at 25 °C prior to taking spectra.

### Far-UV CD and HHP-CD

Far-UV circular dichroism (CD) measurements were performed on a Jasco spectropolarimeter (J-715) at 25 °C. The set-up for spectrum acquisition was scanning from 190–260 nm, resulting in an average of three spectra with a scanning speed of 50 nm min^−1^ and 0.2 nm data steps. Data are shown as raw ellipticity in millidegrees (mdeg) and were acquired using a 0.01-cm path length circular cuvette. α-Syn fibrils were incubated under different HHP conditions (1 h at 516 or 1,033 bar or 10 h at 516 bar) and analyzed after returning to atmospheric pressure. For real-time HHP-CD measurements, a modified HHP unit (ISS model HP-200) was used as previously described^55^. In this modified cell, the optical window was changed from sapphire to MgF_2_ and the aperture diameter reduced from 10 to 3 mm to support measurements in the UV-visible range under pressures up to 2.5 kbar without fracturing^55^. The set-up for HHP-CD was the same as the one used for atmospheric pressure measurements except for the cuvette. In this case, we used a 1-mm path length squared cuvette modified to 20 mm in length for use in the HHP unit. We used a thin strip of parafilm to seal the whole cuvette on its longitudinal axis before sample loading. Then, the sample (ca. 300–400 μL) was loaded using a 1 mL syringe, and an additional strip of parafilm was used to seal the hole formed by the needle. The α-syn fibrils were subjected to 516 bar, and the signal response was followed over time.

### HHP-FTIR

The infrared spectra of α-syn fibrils were recorded and analyzed between 1,660 and 1,550 cm^−1^, corresponding to the amide I’ band region of 1,700–1,600 cm^−1^. Due to the ^13^C and ^15^N isotope labeling, the spectra are shifted 45–50 cm^−1^ to lower wavenumbers^56^,^57^. The amide I′ band is essentially associated with C=O and C–N stretching vibrations, plus C–C–N deformation vibrations of amino-acids. The pressure-dependent FTIR data were recorded using a Nicolet Magna 550 spectrometer, equipped with a liquid-nitrogen-cooled MCT detector. Each FTIR spectrum was obtained by recording 256 scans at a spectral resolution of 2 cm^−1^. The infrared light was focused onto the pinhole of a gas-membrane-driven diamond anvil cell with a brass spacer (Diacell^®^ VivoDAC, Almaxeasy Lab). Fine BaSO_4_ powder was used as an internal pressure calibrant. The sample concentration was 30 mg mL^−1^ in 6 μL of pure D_2_O (no washing steps for this experiment), and using this volume in the DAC cell yielded a sufficiently high signal-to-noise ratio. An external water thermostat served as a temperature control to maintain a temperature of 25 °C. After each pressure change, the sample was equilibrated for 5 min before collection of the IR spectrum. Spectral evaluation was performed using the Thermo GRAMS software as described elsewhere^58^.

Experimental data were fitted using the Voigt function in OriginPro 9.0 G software. Only the bands present in both 2^nd^ derivative and Fourier self-deconvolution (FSD) were used. Peaks were allowed to move ± 2 cm^−1^ and peak widths were limited to 20 cm^−1^. Seven bands were analyzed: 1571, 1580, 1588, 1602, 1612, 1624 and, 1638 cm^−1^. The 1612 band is attributed to α-helix; 1580, 1588 and, 1638 to β-sheet; 1602 to random coils; and 1624 to turns and bends. The 1571 band is attributed to amino-acid side chain vibrations and therefore was omitted from the final plot. However, it was used during the peak-fitting analysis as a baseline correction band.

### Small-angle scattering data collection and analysis

Small angle X-ray scattering (SAXS) studies were performed using the scattering beamline of the National Synchrotron Light Laboratory (LNLS, Campinas, Brazil). We used a 300 K Pilatus detector, 84 mm × 107 mm (Dectris), a mica sample cell holder for liquids at 25 °C, and a wavelength of 1.55 Å. The direct beam position in the detector was calibrated using silver behenate^59^. The sample-detector distance was set to 1,537.945 mm, enabling detection over the *s* range of 0.01–0.2 Å^−1^. X-ray photons elastically scattered from α-syn monomers at different concentrations (140, 500, and 915 μM) or from fibrils after 1 h treatment at 1, 516, 1,033, or 2,067 bars were taken from the average of three frames of 50 sec each, at intervals of 10 sec. The modulus of the scattering vector *s* was calculated according to the equation *s* = (4π/*λ*) sin(2θ), where *λ* is the wavelength used and 2*θ* the scattering angle. The buffer contribution (10 mM Tris-Cl pH 7.4 containing 100 mM NaCl) was subtracted from the sample scattering. Primary processing to obtain Guinier (LnI *vs. s*^2^) and Kratky plots (*s*^2^*I*(*s) vs. s*)^60^ was performed using the PrimusQt suite (ATSAS 2.5 package). The radius of gyration (*R*_g_) from α-syn monomers was determined from the slope (α) of the low-*s* region (*s* < 1.3/*R*_g_) in Guinier plots using the equation α = (*R*_g_^0.5^)/3. Samples were prepared as described in the preparation section and centrifuged at 10,000 *g* for 10 min, 4 °C prior to data acquisition.

### Ensemble optimization method (EOM)

For polydisperse or flexible systems, each conformer makes a scattering contribution to the final scattering profile. The average scattering intensity [*I*(*s*)] is represented by equation (1):


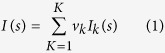


where *v*_*k*_ and *I*_*k*_(*s*) are the volume and the scattering intensity, respectively, from the *k*-th conformers. For very flexible or intrinsically disordered proteins such as α-syn, the deconvolution of scattering data cannot be clearly rationalized due to the large number of solutions, and an ensemble approach is thus the method of choice to interpret scattering data^61^. Based on the input of the amino-acid composition, EOM (*i*) generates a large pool of disordered conformers (pool = 10,000) that approximate the conformational space, (*ii*) computes the theoretical scattering profiles for each conformer using the CRYSOL suite^62^ and (*iii*) using a Genetic algorithm (GA) implemented in the GAJOE suite, selects the subset of conformers that best fits the collected experimental data and minimizes the discrepancies (χ^2^) as follows by equation (2):


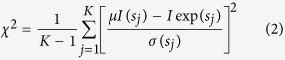


where Iexp (*s*) is the experimental scattering, *K* is the number of experimental points, σ(*s*_*j*_) are the standard deviations, and *μ* is a scaling factor^61^. Scattered samples of α-syn monomers and α-syn species released from fibrils after HHP treatment (2,067 bar) were subjected to EOM analysis. Three GA trials were taken, and very low *s* points (judged by Guinier plots) representing less than 5–8% of the whole scattering curve were depleted. The *R*_g_ and *D*_max_ distributions for each condition are expressed as avg. ± s.d. from the three GA trials.

### NMR assignment of α-syn monomers and HHP-NMR spectroscopy

For assignment, heteronuclear ^1^H-^15^N HSQC NMR spectra were acquired at 288 K using a Bruker Avance III 800-MHz spectrometer. We used α-syn monomers at 600 μM in 10 mM Tris-Cl (pH 7.4), 100 mM NaCl containing 10% D_2_O. ^1^H chemical shifts were referenced to the signal of DSS (diluted to 1 mM in the sample), and ^15^N chemical shifts were indirectly referenced to DSS using a scaling factor of 0.104 obtained from the ^1^H, ^15^N gyromagnetic ratio (γ_H_/γ_N_). The numbers of increment points used were 1,024 for the ^1^H dimension and 702 for the ^15^N dimension, with 2 scans at each increment. We used the ^1^H-^15^N correlations from the biological magnetic resonance data bank - BMRB (BMRB accession number 16543) to aid in the monomer assignment process. We were able to analyze changes in 100 of 140 ^1^H-^15^N correlations by NMR.

For the HHP-NMR experiments, we used the same set-up as described above, and the spectra of α-syn monomers and fibrils were acquired using a zirconia NMR tube with an internal diameter of 3 mm and an outer diameter of 5 mm (Daedalus Innovations). An Xtreme-60 syringe pump system (Daedalus Innovations) was used to generate pressure increments from 1 to 250, 500, 750, 1,000, 1,250, 1,500, 1,750, 2,250, and 2,500 bar. At each pressure increment, the sample was left for 20 min for accommodation before spectrum acquisition. The NMR assignment of the α-syn monomer HSQC spectra at different pressures was performed by tracking the systematic shifts using the assigned HSQC at 1 bar. This step was important to exclude the compressibility contribution of pressure on monomers and to aid in finding the ^1^H-^15^N correlations of monomeric fractions released from fibrils.

For line-broadening analysis, the relative intensity of each correlation obtained for the α-syn monomers and monomeric species released from fibrils was plotted as a function of pressure increase. The monomeric species released from the fibrils presented a bell-shaped behavior and were fitted to a second-order polynomial equation (y = bx^2^ +  ax + c, where y is relative intensity and x is pressure) using the Origin 8.0 software (Northampton, MA, USA).

Chemical shift perturbation (CSP) analysis of the α-syn monomers was performed at 500, 1,000, and 2,500 bar against ^1^H-^15^N HSQC NMR spectra at 1 bar using equation (3):


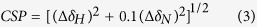


where Δδ_H_ and Δδ_N_ represent the chemical shift variations of ^1^H and ^15^N, respectively, between different pressure increments (500, 1,000, and 2,500) and 1 bar. All spectra were processed using Topspin 3.11, and chemical shift values were measured using the CCPN Analysis 2.4.1 suite^63^.

### CPMG-RD measurements

Carr-Purcell-Meiboom-Gill relaxation dispersion (CPMG-RD) measurements^64^ at mild pressures (500 and 750 bar) were acquired on a Bruker Avance III 800-MHz spectrometer at 288 K. Relaxation rates (R_2 eff_) were calculated from the intensities of resonance in the^1^H-^15^N correlation spectra acquired with two CPMG frequencies (υ_CPMG_ 50 and 1000 Hz) using equation (4):


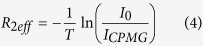


where T is time and I_0_ and I_CPMG_ are the intensities of resonance obtained from experiments without and with CPMG pulse block. R_2eff_ at υ_CPMG_ of 50 and 1000 Hz was plotted as a function of α-syn residue to probe residues experiencing conformational exchange motions. Samples were kept at the specified pressure for 20 min for accommodation before CPMG-RD measurements.

### Solid-state NMR spectroscopy

The experiments were performed in a standard bore with a static magnetic field of 700 MHz (Bruker, Avance III) using a 3.2 mm triple resonance (^1^H/^13^C/^15^N) and flip angle probe head at the National Center of Nuclear Magnetic Resonance, RJ, Brazil, and in a wide bore with a static magnetic field of 600 MHz (Bruker, Avance III) using a 4.0 mm triple resonance probe head at the Leibniz Institute für Molekulare Pharmakologie, Berlin, Germany. The magic angle was calibrated with KBr^79^, the field homogeneity was established using adamantane, and the line width approximately 3 Hz was obtained. The sample temperature was calculated as indicated previously^65^. In all experiments using the 3.2 mm probe head, the temperature was approximately 10 °C, and the spinning speed was 14 kHz. With 4.0 mm rotors, the thermostat was set to 2 °C. For ^13^C–^13^C correlation spectra, a DARR^66^ mixing period of 20 ms was applied using an RF spinal64 of 85 kHz. The recovery delay was 3 sec because the global T1 was 890 ms. The data processing was performed in a Topspin 3.2pl6 (Bruker) with squared sine bell (qsine 3) apodization as a window function in the indirect dimensions and Gaussian in the direct dimension, and a four-fold zero filling was applied in the indirect and direct dimensions. In all experiments, the data were analyzed using the CCPN NMR software^63^.

### Seeding experiments

Fibrillation reactions were performed using 140 μM of α-syn monomers diluted in 10 mM Tris-Cl, 100 mM NaCl pH 7.4 and monitored by the fluorescence of 10 μM ThT. We recorded emission points at ThT *λ*_max_ (i.e., 477 nm) upon excitation at 450 nm. We used a black-bottom 96-well plate (Thermo Scientific) with a final reaction volume of 100 μL in each well. Plates were sealed with clear polyolefin sealing tape (Fisher Scientific), loaded into a SpectraMax Paradigm Multi-Mode Microplate reader (Molecular Devices) and incubated at 37 °C. Samples were subjected to orbital agitation. ThT emission was measured at 4 min intervals for 20 h, while the ThT signal stabilized. To generate different α-syn seeds, equal amounts of washed fibrils from the same fibrillation reaction (ca. 75–80 μM) were prepared to a final volume of 1 mL. The seeds were generated after incubation for 1 h at 1,033 bar or 2,067 bar (SMMs + remaining fibrils [Rm-F]). Preformed seeds were generated by subjecting fibrils to a sonication pulse of 10 sec using 50% power amplification (Sonics&Materials, Inc.). α-Syn seeds in the concentration ranges of 1 to around 40 μg, in a final reaction volume of 100 μL/well were incubated with 140 μM α-syn monomers.

### Computational analysis

Internal cavities and surface clefts were mapped in the ssNMR structure of the α-syn fibril [Protein Data Bank (PDB) ID code 2n0a] based on the Monte Carlo method included in the McVol suite^67^. The applied algorithm used a probe sphere of 1.3 Å, 50 Monte Carlo steps per Å^3^ of the molecule, and 2,500 dots per atom on the dotted surface. The minimum volume for cavities to be considered was 7 Å. The identified cavities were visualized using PyMOL.

## Additional Information

**How to cite this article**: de Oliveira, G. A. P. *et al*. Structural basis for the dissociation of α-synuclein fibrils triggered by pressure perturbation of the hydrophobic core. *Sci. Rep.* **6**, 37990; doi: 10.1038/srep37990 (2016).

**Publisher's note:** Springer Nature remains neutral with regard to jurisdictional claims in published maps and institutional affiliations.

## Supplementary Material

Supplementary Information

## References

[b1] Spillantini M. G. . Alpha-synuclein in Lewy bodies. Nature 388, 839–840 (1997). 9278044 10.1038/42166

[b2] Polymeropoulos M. H. . Mutation in the alpha-synuclein gene identified in families with Parkinson’s disease. Science 276, 2045–2047 (1997). 9197268 10.1126/science.276.5321.2045

[b3] Krüger R. . Ala30Pro mutation in the gene encoding alpha-synuclein in Parkinson’s disease. Nat. Genet. 18, 106–108 (1998). 9462735 10.1038/ng0298-106

[b4] Singleton A. B. . alpha-Synuclein locus triplication causes Parkinson’s disease. Science 302, 841 (2003). 14593171 10.1126/science.1090278

[b5] Chartier-Harlin M. C. . Alpha-synuclein locus duplication as a cause of familial Parkinson’s disease. Lancet 364, 1167–1169 (2004). 15451224 10.1016/S0140-6736(04)17103-1

[b6] Maraganore D. M. . High-resolution whole-genome association study of Parkinson disease. Am. J. Hum. Genet. 77, 685–693 (2005). 16252231 10.1086/496902 PMC1271381

[b7] Lee H. J., Choi C. & Lee S. J. Membrane-bound alpha-synuclein has a high aggregation propensity and the ability to seed the aggregation of the cytosolic form. J. Biol. Chem. 277, 671–678 (2002). 11679584 10.1074/jbc.M107045200

[b8] Abeliovich A. . Mice lacking α-synuclein display functional deficits in the nigrostriatal dopamine system. Neuron 25, 239–252 (2000). 10707987 10.1016/s0896-6273(00)80886-7

[b9] Nemani V. M. . Increased expression of alpha-synuclein reduces neurotransmitter release by inhibiting synaptic vesicle reclustering after endocytosis. Neuron 65, 66–79 (2010). 20152114 10.1016/j.neuron.2009.12.023 PMC3119527

[b10] Burré J. . Alpha-synuclein promotes SNARE-complex assembly *in vivo* and *in vitro*. Science 329, 1663–1667 (2010). 20798282 10.1126/science.1195227 PMC3235365

[b11] Fauvet B. . α-Synuclein in the central nervous system and from erythrocytes, mammalian cells and *E. coli* exists predominantly as a disordered monomer. J. Biol. Chem. 287, 15345–15364 (2012). 22315227 10.1074/jbc.M111.318949 PMC3346117

[b12] Theillet F.-X. . Structural disorder of monomeric α-synuclein persists in mammaliam cells. Nature 530, 45–50 (2016). 26808899 10.1038/nature16531

[b13] Bartels T., Choi J. G. &Selkoe D. J. α-Synuclein occurs physiologically as a helically folded tetramer that resists aggregation. Nature 477, 107–110 (2011). 21841800 10.1038/nature10324 PMC3166366

[b14] Wang W. . A soluble α-synuclein construct forms a dynamic tetramer. Proc.Natl Acad. Sci. USA 108, 17797–17802 (2011). 22006323 10.1073/pnas.1113260108 PMC3203798

[b15] Davidson W. S., Jonas A., Clayton D. F. & Georges J. M. Stabilization of α-synuclein secondary structure upon binding to synthetic membranes. J. Biol. Chem. 273, 9443–9449 (1998). 9545270 10.1074/jbc.273.16.9443

[b16] Bartels T. . The N-terminus of the intrinsically disordered protein α-synuclein triggers membrane binding and helix folding. Biophys. J. 99, 2116–2124 (2010). 20923645 10.1016/j.bpj.2010.06.035 PMC3042581

[b17] Fusco G. . Direct observation of the three regions in α-Synuclein that determine its membrane-bound behaviour. Nat.Commun. 5, 3827 (2014). 24871041 10.1038/ncomms4827 PMC4046108

[b18] Galvagnion C. . Lipid vesicles trigger α-synuclein aggregation by stimulating primary nucleation. Nat. Chem. Biol. 11, 229–234 (2015). 25643172 10.1038/nchembio.1750 PMC5019199

[b19] Dehay B. . Targeting α-synuclein for treatment of Parkinson’s disease: mechanistic and therapeutic considerations. Lancet Neurol. 14, 855–866 (2015). 26050140 10.1016/S1474-4422(15)00006-X PMC5217462

[b20] Silva J. L. . High-pressure chemical biology and biotechnology. Chem. Rev. 114, 7239–7267 (2014). 24884274 10.1021/cr400204z

[b21] Hummer G., Garde S., García A. E., Paulaitis M. E. & Pratt L. R. The pressure dependence of hydrophobic interactions is consistent with the observed pressure denaturation of proteins. Proc. Natl Acad. Sci. USA 95, 1552–1555 (1998). 9465053 10.1073/pnas.95.4.1552 PMC19087

[b22] Oliveira A. C., Gaspar L. P., Da Poian A. T. & Silva J. L. Arc repressor will not denature under pressure in the absence of water. J. Mol. Biol. 240, 184–187 (1994). 8028002 10.1006/jmbi.1994.1433

[b23] Foguel D. . Dissociation of amyloid fibrils of α-synuclein and transthyretin by pressure reveals their reversible nature and the formation of water-excluded cavities. Proc.Natl Acad. Sci. USA 100, 9831–9836 (2003). 12900507 10.1073/pnas.1734009100 PMC187855

[b24] Follmer C. . Dopamine affects the stability, hydration, and packing of protofibrils and fibrils of the wild type and variants of α-synuclein. Biochemistry 46, 472–482 (2007). 17209557 10.1021/bi061871+

[b25] Li J. Y. . Lewy bodies in grafted neurons in subjects with Parkinson’s disease suggest host-to-graft disease propagation. Nat. Med. 14, 501–503 (2008). 18391963 10.1038/nm1746

[b26] Desplats P. . Inclusion formation and neuronal cell death throughneuron-to-neuron transmission of alpha-synuclein. Proc.Natl Acad. Sci. USA 106, 13010–13015 (2009). 19651612 10.1073/pnas.0903691106 PMC2722313

[b27] Luk K. C. . Exogenous α-synuclein fibrils seed the formation of Lewy body-like intracellular inclusions in cultured cells. Proc.Natl Acad. Sci. USA 106, 20051–20056 (2009). 19892735 10.1073/pnas.0908005106 PMC2785290

[b28] Hansen C. . alpha-Synuclein propagates from mouse brain to grafted dopaminergic neurons and seeds aggregation in cultured human cells. J.Clin. Invest. 121, 715–725 (2011). 21245577 10.1172/JCI43366 PMC3026723

[b29] Luk K. C. . Pathological α-synuclein transmission initiates Parkinson-like neurodegeneration in nontransgenic mice. Science 338, 949–953 (2012). 23161999 10.1126/science.1227157 PMC3552321

[b30] Prusiner S. B. . Evidence for α-synuclein prions causing multiple system atrophy in humans with parkinsonism. Proc.Natl Acad. Sci. USA 112, E5308–5317 (2015). 26324905 10.1073/pnas.1514475112 PMC4586853

[b31] Tuttle M. D. . Solid-state NMR structure of a pathogenic fibril of full-length human α-synuclein. Nat. Struct. Mol. Biol. 23, 409–415 (2016). 27018801 10.1038/nsmb.3194 PMC5034296

[b32] Roche J. . Cavities determine the pressure unfolding of proteins. Proc.Natl Acad. Sci. USA 109, 6945–6950 (2012). 22496593 10.1073/pnas.1200915109 PMC3344970

[b33] de Oliveira G. A. & Silva J. L. A hypothesis to reconcile the physical and chemical unfolding of proteins. Proc.NatlAcad.Sci. USA 112, E2775–2784 (2015). 10.1073/pnas.1500352112 PMC4450381 25964355

[b34] Bellissent-Funel M. C. . Water determines the structure and dynamics of proteins. Chem. Rev. 116, 7673–7697 (2016). 27186992 10.1021/acs.chemrev.5b00664 PMC7116073

[b35] Chen S. W. . Structural characterization of toxic oligomers that are kinetically trapped during α-synuclein fibril formation. Proc.Natl Acad. Sci. USA 112, E1994–2003 (2015). 25855634 10.1073/pnas.1421204112 PMC4413268

[b36] Kayed R. . Common structure of soluble amyloid oligomers implies common mechanism of pathogenesis. Science 300, 486–489 (2003). 12702875 10.1126/science.1079469

[b37] Akasaka K. & Li H. Low-lying excited states of proteins revealed from nonlinear pressure shifts in 1H and 15N NMR. Biochemistry 40, 8665–8671 (2001). 11467925 10.1021/bi010312u

[b38] Kitahara R. . A delicate interplay of structure, dynamics, and thermodynamics for function: a high pressure NMR study of outer surface protein A. Biophys. J. 102, 916–926 (2012). 22385863 10.1016/j.bpj.2011.12.010 PMC3283806

[b39] Peng X., Jonas J. & Silva J. L. Molten-globule conformation of Arc repressor monomers determined by high-pressure 1H NMR spectroscopy. Proc. Natl Acad. Sci. USA 90, 1776–1780 (1993). 8446590 10.1073/pnas.90.5.1776 PMC45962

[b40] Heise H. . Molecular-level secondary structure, polymorphism, and dynamics of full-length alpha-synuclein fibrils studied by solid-state NMR. Proc. Natl Acad. Sci.USA 102, 15871–15876 (2005). 16247008 10.1073/pnas.0506109102 PMC1276071

[b41] Kloepper K. D., Hartman K. L., Ladror D. T. & Rienstra C. M. Solid-state NMR spectroscopy reveals that water is nonessential to the core structure of alpha-synuclein fibrils. J. Phys. Chem. B 111, 13353–13356 (2007). 17985869 10.1021/jp077036z PMC2551327

[b42] Golebiewska U. & Scarlata S. High pressure promotes alpha-synuclein aggregation in cultured neuronal cells. FEBS Lett. 589, 3309–3312 (2015). 26434717 10.1016/j.febslet.2015.09.019 PMC4661088

[b43] Calamai M. . Reversal of protein aggregation provides evidence for multiple aggregated states. J. Mol. Biol. 346, 603–616 (2005). 15670608 10.1016/j.jmb.2004.11.067

[b44] MacPhee C. E. & Dobson C. M. Chemical dissection and reassembly of amyloid fibrils formed by a peptide fragment of transthyretin. J. Mol. Biol. 297, 1203–1215 (2000). 10764584 10.1006/jmbi.2000.3600

[b45] Cremades N. . Direct observation of the interconversion of normal and toxic forms of α-synuclein. Cell 149, 1048–1059 (2012). 22632969 10.1016/j.cell.2012.03.037 PMC3383996

[b46] El-Agnaf O. M. . Alpha-synuclein implicated in Parkinson’s disease is present in extracellular biological fluids, including human plasma. FASEB J. 17, 1945–1947 (2003). 14519670 10.1096/fj.03-0098fje

[b47] Ruschak A. M. & Miranker A. D. Fiber-dependent amyloid formation as catalysis of an existing reaction pathway. Proc. Natl Acad. Sci. USA 104, 12341–12346 (2007). 17640888 10.1073/pnas.0703306104 PMC1941471

[b48] Knowles T. P. . An analytical solution to the kinetics of breakable filament assembly. Science 326, 1533–1537 (2009). 20007899 10.1126/science.1178250

[b49] Cohen S. I. . Proliferation of amyloid-β42 aggregates occurs through a secondary nucleation mechanism. Proc. Natl Acad. Sci. USA 110, 9758–9763 (2013). 23703910 10.1073/pnas.1218402110 PMC3683769

[b50] Buell A. K. . Solution conditions determine the relative importance of nucleation and growth processes in α-synuclein aggregation. Proc. Natl Acad. Sci. USA 111, 7671–7676 (2014). 24817693 10.1073/pnas.1315346111 PMC4040554

[b51] Pronchik J., He X., Giurleo J. T. & Talaga D. S. *In vitro* formation of amyloid from alpha-synuclein is dominated by reactions at hydrophobic interfaces. J. Am. Chem. Soc. 132, 9797–9803 (2010). 20578692 10.1021/ja102896h

[b52] Ferrão-Gonzales A. D., Souto S. O., Silva J. L. & Foguel D. The preaggregated state of an amyloidogenic protein: hydrostatic pressure converts native transthyretin into the amyloidogenic state. Proc. Natl Acad. Sci. USA 97, 6445–6450 (2000). 10841549 10.1073/pnas.97.12.6445 PMC18622

[b53] Coelho-Cerqueira E., Carmo-Gonçalves P., SáPinheiro A., Cortines J. & Follmer C. α-synuclein as an intrinsically disordered monomer - fact or artefact? FEBS J. 280, 4915–4927 (2013). 23927048 10.1111/febs.12471

[b54] Tugarinov V., Kanelis V. & Kay L. E. Isotope labeling strategies for the study of high-molecular-weight proteins by solution NMR spectroscopy. Nat.Protoc. 1, 749–754 (2006). 17406304 10.1038/nprot.2006.101

[b55] Lerch M. T., Horwitz J., McCoym J. & Hubbell W. L. Circular dichroism and site-directed spin labeling reveal structural and dynamical features of high-pressure states of myoglobin. Proc.Natl Acad. Sci. USA 110, E4714–E4722 (2013). 24248390 10.1073/pnas.1320124110 PMC3856799

[b56] Haris P. I., Robillard G. T., van Dijk A. A. & Chapman D. Potential of 13C and 15N labeling for studying protein-protein interactions using Fourier transform infrared spectroscopy. Biochemistry 31, 6279–6284 (1992). 1320934 10.1021/bi00142a016

[b57] Li T. Investigation of protein-protein interactions by isotope-edited Fourier transformed infrared spectroscopy. Spectroscopy 18, 397–406 (2004).

[b58] Panick G. . Structural characterization of the pressure-denatured state and unfolding/refolding kinetics of staphylococcal nuclease by synchrotron small-angle X-ray scattering and Fourier-transform infrared spectroscopy. J. Mol. Biol. 275, 389–402 (1998). 9466917 10.1006/jmbi.1997.1454

[b59] Huang T. C., Toraya H., Blanton T. N. & Wu Y. X-ray powder diffraction analysis of silver behenate, a possible low-angle diffraction standard. J. Appl.Cryst. 26, 180–184 (1993).

[b60] Doniach S. Changes in biomolecular conformation seen by small angle X-ray scattering. Chem. Rev. 101, 1763–1778 (2001). 11709998 10.1021/cr990071k

[b61] Bernadó P., Mylonas E., Petoukhov M. V., Blackledge M. & Svergun D. I. Structural characterization of flexible proteins using small-angle X-ray scattering. J. Am. Chem. Soc. 129, 5656–5664 (2007). 17411046 10.1021/ja069124n

[b62] Svergun D. I., Barberato C. & Koch M. H. J. Crysol - a program to evaluate X-ray solution scattering of biological macromolecules from atomic coordinates. J. Appl.Cryst. 28, 768–773 (1995).

[b63] Vranken W. F. . The CCPN data model for NMR spectroscopy: development of a software pipeline. Proteins 59, 687–696 (2005). 15815974 10.1002/prot.20449

[b64] Carr H. Y. & Purcell E. M. Effects of diffusion on free precession in nuclear magnetic resonance experiments. Phys. Rev. 94, 630–638 (1954).

[b65] Böckmann A. . Characterization of different water pools in solid-state NMR protein samples. J. Biomol. NMR 45, 319–327 (2009). 19779834 10.1007/s10858-009-9374-3

[b66] Takegoshi K., Nakamura S. & Terao T. 13C-1H dipolar-assisted rotational resonance in magic-angle spinning NMR. Chem. Phys.Lett. 344, 631–637 (2001).

[b67] Till M. S. & Ullmann G. M. McVol - a program for calculating protein volumes and identifying cavities by a Monte Carlo algorithm. J. Mol. Model. 16, 419–429 (2010). 19626353 10.1007/s00894-009-0541-y

