# Supplementary material for: Structural basis for the dissociation of α-synuclein fibrils triggered by pressure perturbation of the hydrophobic core
Source: Sci Rep. 2016 Nov 30;6:37990. doi: 10.1038/srep37990 (PMC5128797; doi:10.1038/srep37990)
Supplement: Supplementary Information [file srep37990-s1.doc]

**Structural basis for the dissociation of α-synuclein fibrils triggered by pressure perturbation of the hydrophobic core**

Guilherme A. P. de Oliveira1#, Mayra de A. Marques1#, Carolina Cruzeiro-Silva1#, Yraima Cordeiro2, Caroline Schuabb3, Adolfo H. Moraes1,4, Roland Winter3, Hartmut Oschkinat5, Debora Foguel1, Mônica S. de Freitas1* and Jerson L. Silva1*

1 Programa de Biologia Estrutural, Instituto de Bioquímica Médica Leopoldo de Meis, Instituto Nacional de Biologia Estrutural e Bioimagem, Centro Nacional de Ressonância Magnética Nuclear Jiri Jonas, Universidade Federal do Rio de Janeiro, Rio de Janeiro, Brazil.

2 Faculdade de Farmácia, Universidade Federal do Rio de Janeiro, Rio de Janeiro, Brazil.

3 Department of Chemistry and Chemical Biology, Physical Chemistry, Technische Universität Dortmund, Dortmund, Germany.

4 Departamento de Química, Instituto de Ciências Exatas, Universidade Federal de Minas Gerais, Brazil.

5 Department of Structural Biology, Leibniz Institute für Molekulare Pharmakologie, Berlin, Germany.

**
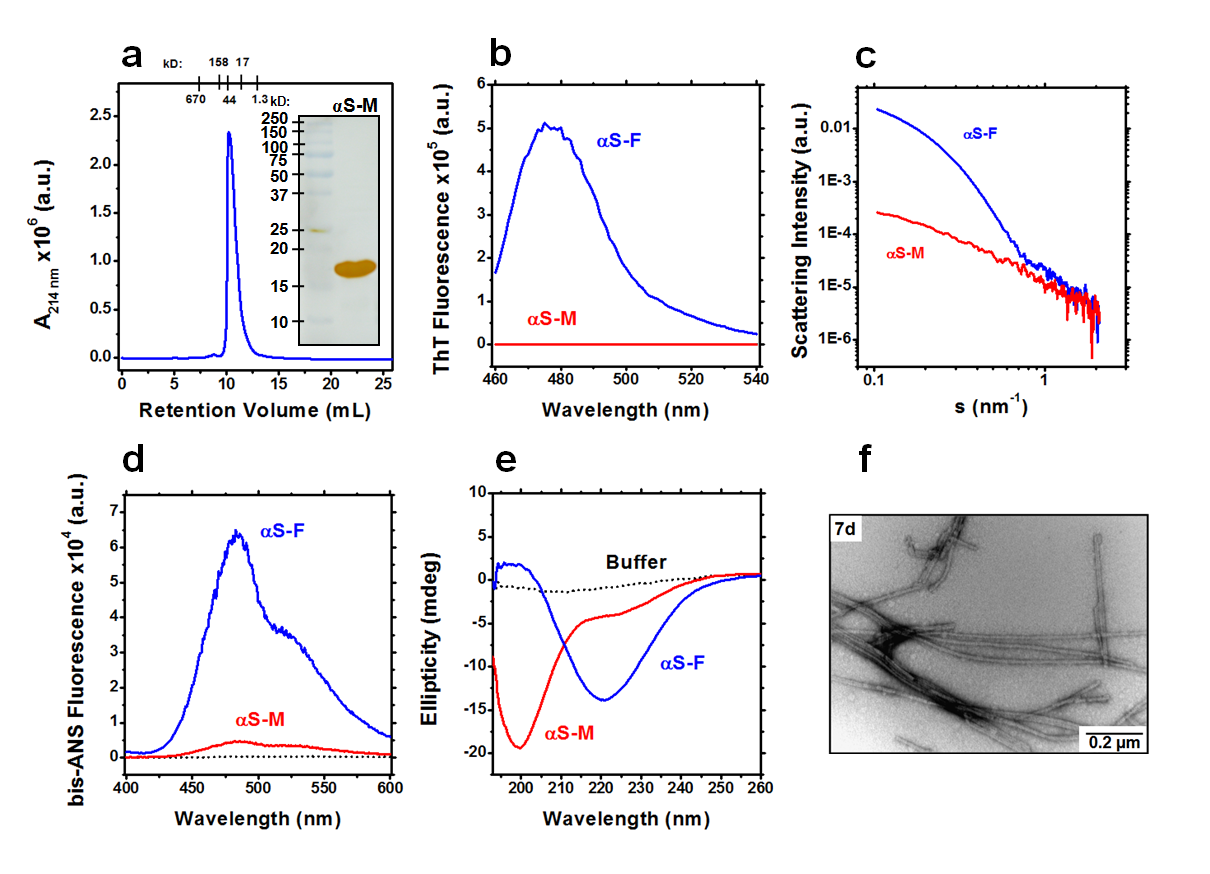
**

**Supplementary Figure 1. Biochemical and spectroscopic characterization of α-syn fibrils. (a)** Size exclusion chromatography of α-syn monomers in GPC-250 column as the final step before fibrillation reaction. Inset shows silver staining of α-syn monomers in 12.5% SDS-PAGE. **(b)** Emission spectrum of ThT signal of equal amounts of α-syn monomers and fibrils. **(c)** SAXS scattering profile of α-syn monomers and fibrils obtained using synchrotron radiation. **(d)** Emission spectrum of bis-ANS binding to α-syn fibrils. **(e)** Far-UV circular dichroism measurements of α-syn monomers and fibrils. **(f)** Negative staining transmission electron microscopy of α-syn fibrils after incubation of 7 days.


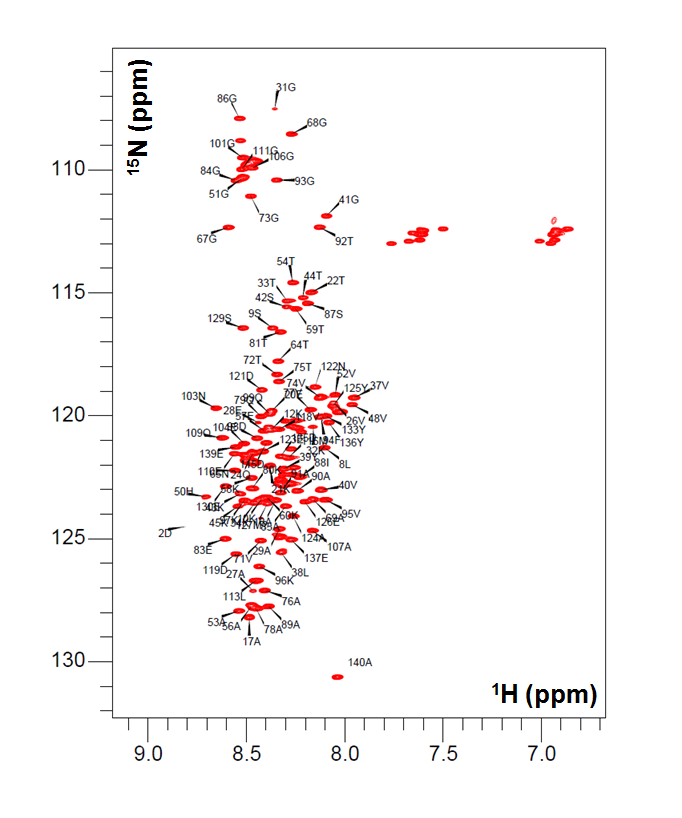


**Supplementary Figure 2. Assignment of α-syn monomer.** 1H-15N HSQC spectrum showing assigned cross peaks. We were able to follow 100 out of 140 1H-15N correlations for the monomeric form of α-syn at 1 bar.

**
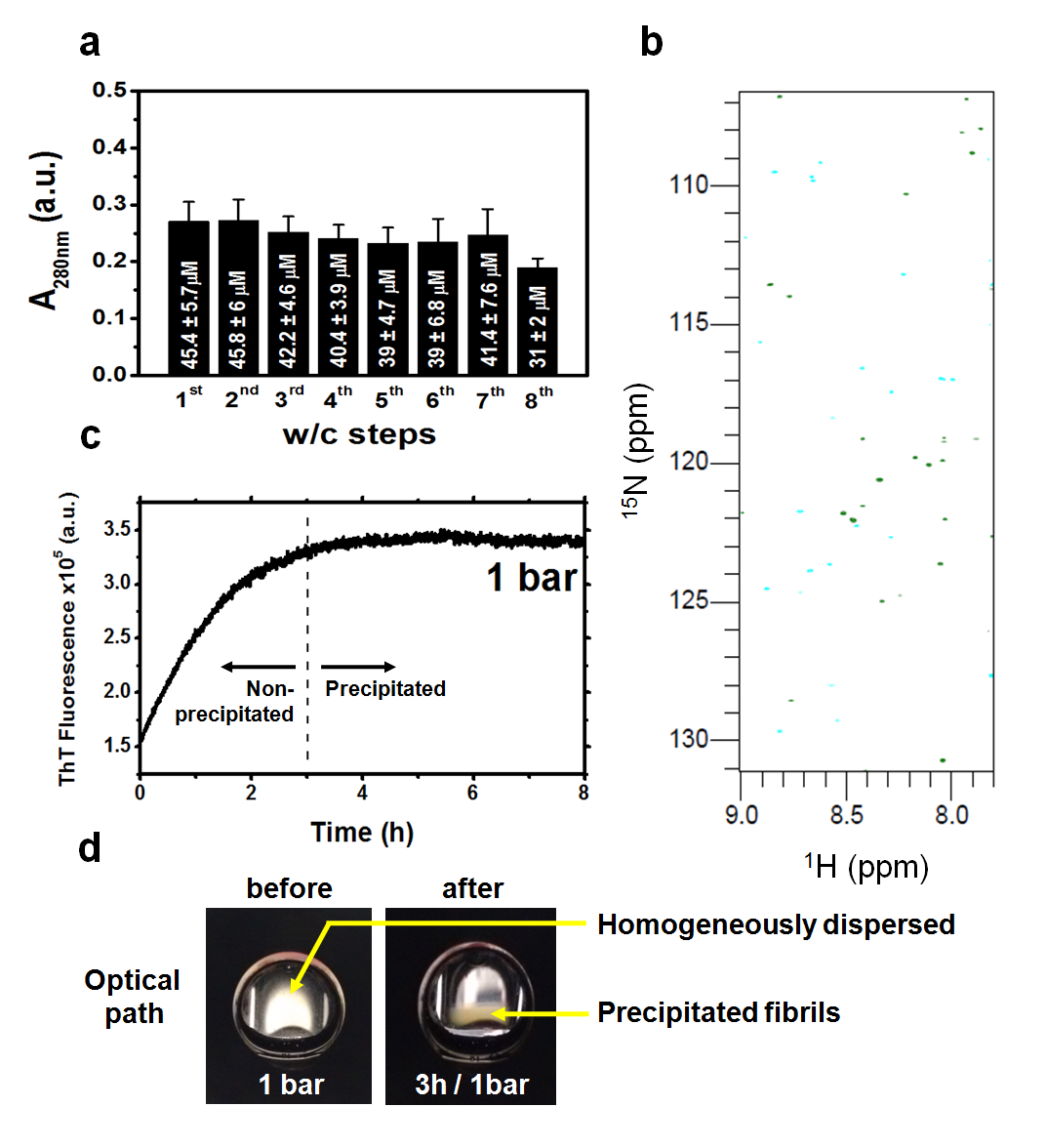
**

**Supplementary Figure 3. Washing / centrifugation (w/c) protocol validation and control experiments for the kinetics of fibril disassembly upon HHP treatment. (a)** Control experiment revealing that our w/c steps do not affect the initial amount of α-syn fibrils due to dissociation to monomers. After the fibrillation reactions has taken place, fibrils were harvested and the w/c steps were performed as described in Methods. After each centrifugation, supernatants were replaced and solutions gently mixed (only by swirling). Following this, an aliquot of 50 µL was taken and harvested at 21,800 g, 5 min, 4 °C. Dried pellets from aliquots were then resuspended in 100 µL of 5 M guanidinium chloride and measured for A_280nm_. Concentrations inside the bars represent the amount of α-syn monomers dissociated from fibrils due to dissolution with 5 M guanidinium chloride at each washing step. Experiments are shown as avg. ± s.e.m. (*n* = 4, independent fibrillation reactions). **(b)** 1H-15N HSQC spectra of α-syn (αS) fibrils at 1bar. The empty fibril spectrum is shown to verify that no remaining monomers were present at the initial HHP titration of fibrils. Green and cyan peaks correspond to noise signals. **(c)** Raw ThT fluorescence data of fibrils at 1 bar, starting from non-sedimented fibrils. The increasing ThT signal from 0 up to 3 h represents the precipitation process of α-syn fibrils through the optical path, and was set to the minimum time required to stabilize the ThT signal and to obtain sedimented fibrils. **(d)** The height of the sample holder was adjusted to guarantee that sedimented fibrils were maintained in the optical path before applying pressure increments. The picture illustrates the front view of the optical path with homogeneously dispersed α-syn fibrils bound to ThT (before) and sedimented fibrils bound to ThT after 3h of rest.

**
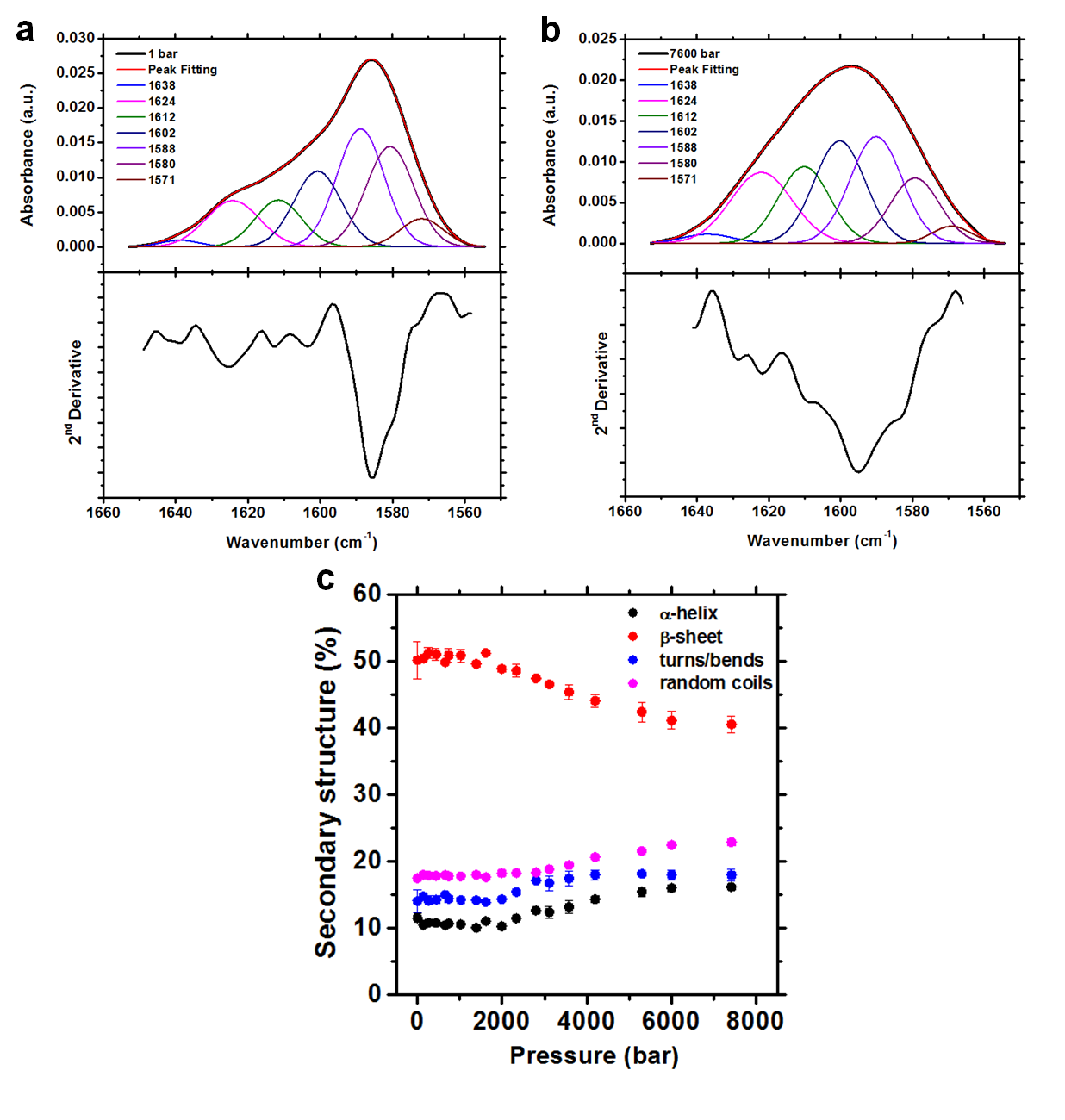
**

**Supplementary Figure 4. HHP-FTIR deconvolution spectra.** Analysis of deconvolution spectra (upper panels) of 15N/13C α-syn fibrils at **(a)** 1 bar and **(b)** 7,600 bar. Negative peaks (black lines) of second derivative curves (bottom panels) are in good agreement with the Gaussian peaks obtained from the fitting of the absorption data. Seven Gaussian peaks were used to describe the conformational space of the populations of α-syn fibrils and the species recovered after sample depressurization. The fits (red lines) are overlapping with the raw data (black lines) and show the good quality of the fits. Because we have used 15N/13C isotope-labeled samples, the absorbance bands of the carbonyl moieties are shifted by ~45 cm-1 56, 57. **(c)** Secondary structure analysis as a function of increasing pressure.


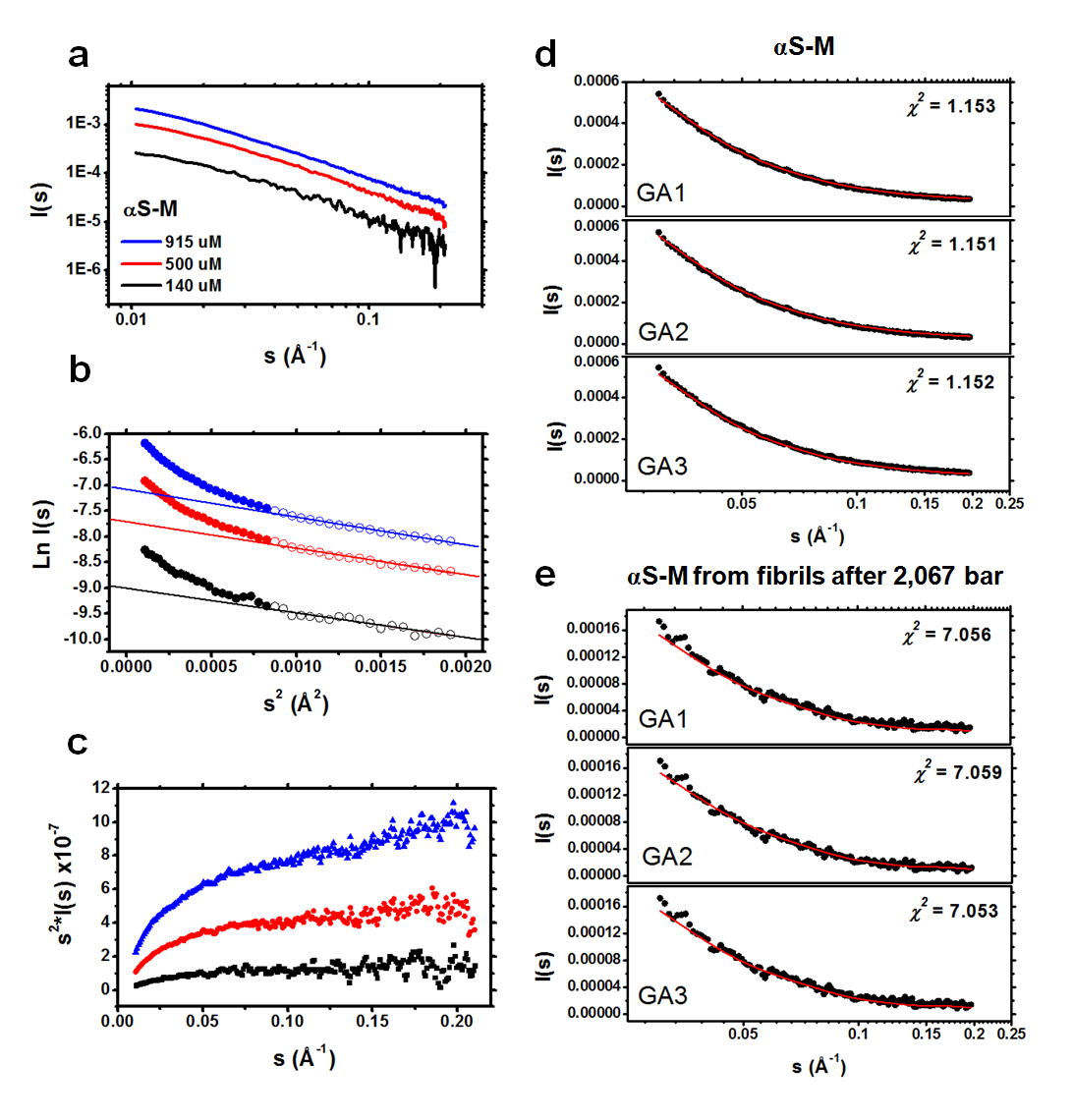


**Supplementary Figure 5. Primary processing of SAXS scattering data for αS-M. and genetic algorithm runs during EOM analysis. (a)** Scattering intensity *I*(*s*) as a function of the scattering vector *s* of α-syn monomers at 140, 500, and 915 µM. **(b)** The low *s* region in the Guinier plots (open spheres) from **(a)** were figured out using values of *s* < 1.3/*R*_g_. The *R*_g_ of α-syn monomers estimated from the Guinier analysis were similar among the concentrations measured: 38.6 ± 10.8 Å for 140 µM, 40.4 ± 8 Å for 500 µM, and 40.8 ± 6.9 Å for 915 µM. **(c)** Kratky plots from **(a)** reveal a monotonic increase in the values of *s*2*I*(*s*) as a function of *s*, indicating unfolding of α-syn monomers in the concentration range covered. The color scheme in a, b and c is the same. **(d, e)** The fits (red lines) for the three genetic algorithm (GA) trials employed during EOM analysis and chi-square values are shown for initial α-syn monomers and species released from fibrils after 1 h at 2,067 bar.


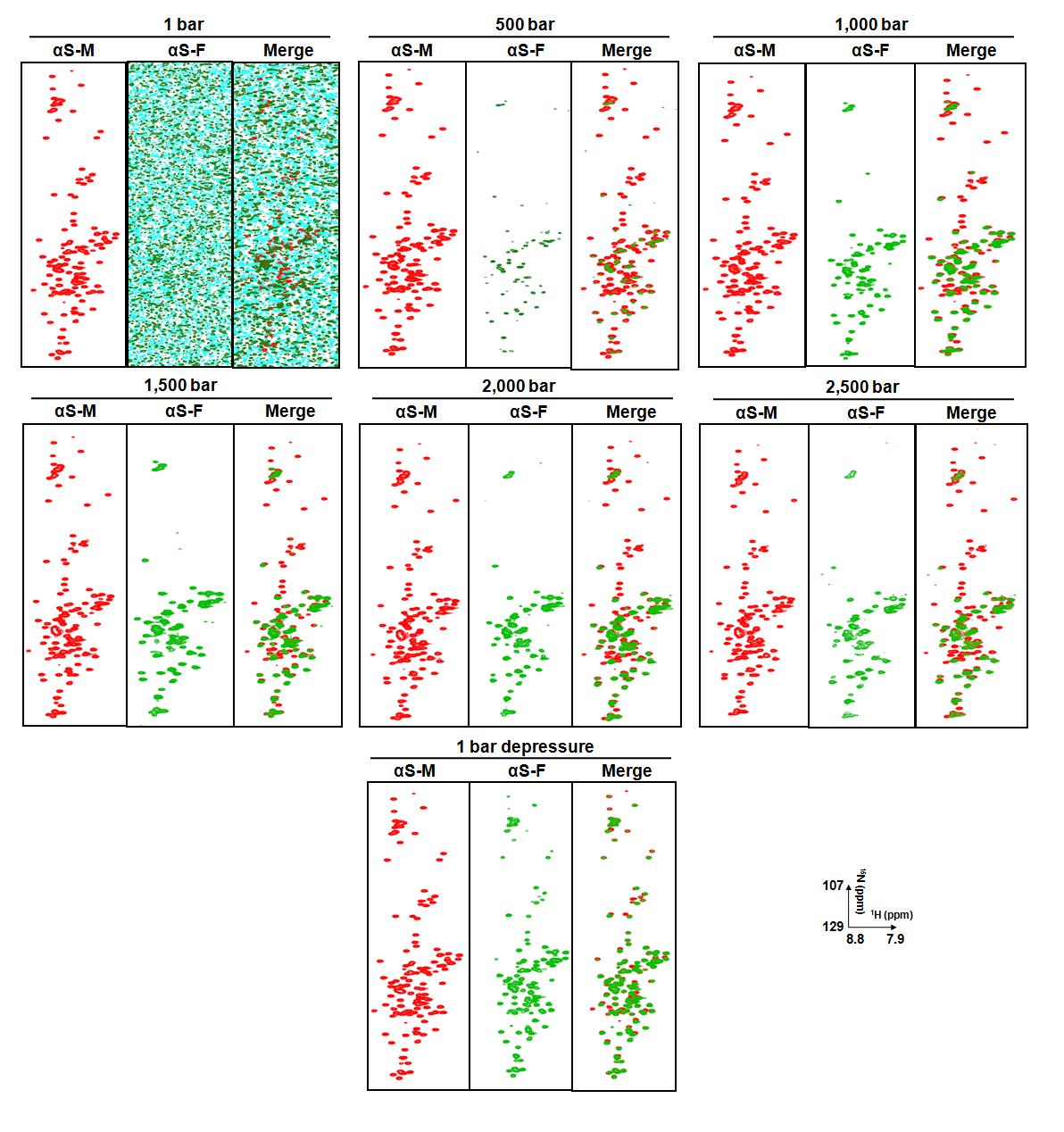


**Supplementary Figure 6. Superposition of 1H-15N HSQC spectra between α-syn monomers and monomeric species released from α-syn fibrils upon HHP.** A collection of HSQC spectra are shown for α-syn monomers (red) and monomeric species released from fibrils (green) at 1, 500, 1,000, 1,500, 2,000, 2,500, and after decompression. Merged spectra are depicted at each pressure point to show how we were able to correlate both species. Minor chemical shifts were observed between initial monomers (red) and monomeric species released from fibrils (green) at each pressure increment, making the assignment tracking possible for each pressure condition.

**
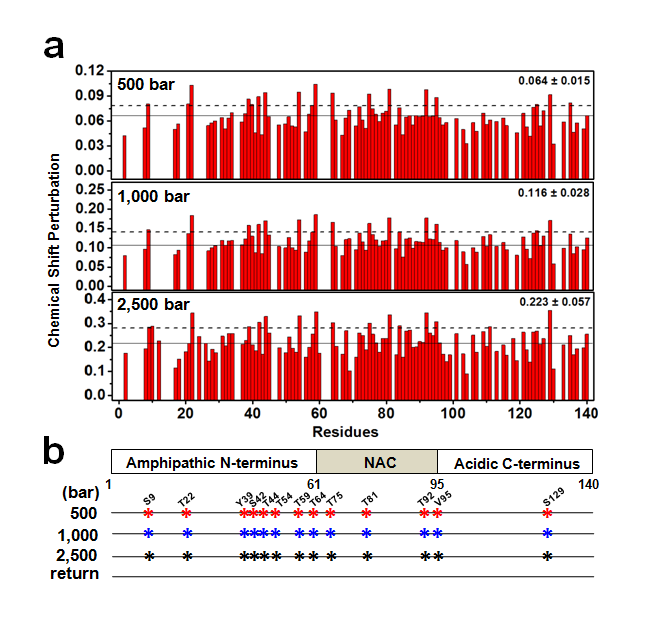
**

**Supplementary Figure 7. Chemical shift perturbation (CSP) analysis for the effects of HHP to α-syn monomers.** CSP analysis were performed at 500, 1,000, and 2,500 bar against 1H-15N HSQC NMR spectra at 1 bar. **(a)** Plots show CSP values as a function of residue number for 500, 1,000, and 2,500 bar. Solid lines show the avg. of CSP values and dashed lines the avg. + 1s.d. among residues. These values were 0.064 ± 0.015 for 500 bar, 0.116 ± 0.028 for 1,000 bar, and 0.223 ± 0.057 for 2,500 bar. **(b)** Residues experiencing CSP values higher than the avg. +1.s.d are depicted at each evaluated pressure. Of note, there was a systematic dependence of some residues to CSP values at increasing pressures revealing the most labile segments of α-syn monomers to the compressibility effects of HHP. Spectra of monomers after decompression were identical to those at 1 bar, indicating that CSP values were zero, as represented by the empty solid line for the return.


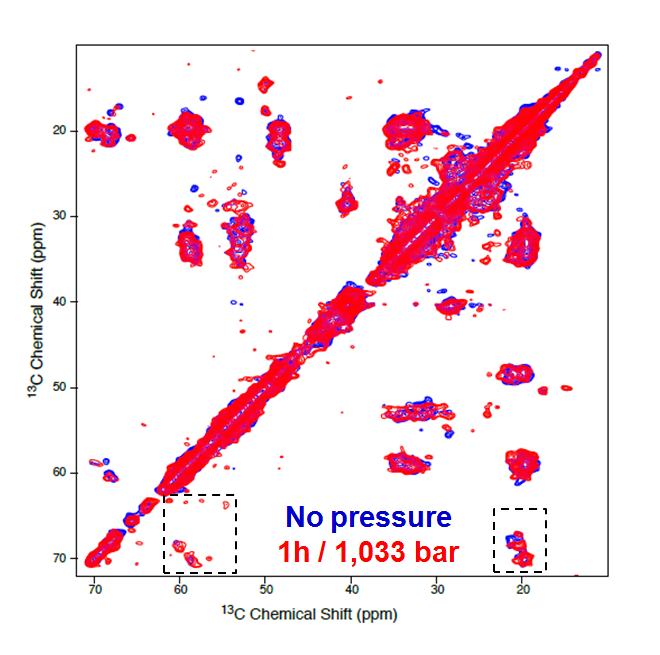


**Supplementary Figure 8.** 13C-13C correlation spectrum acquired on a static magnetic field NMR spectrometer of 700 MHz for the α-syn fibril core before (blue) and after (red) 1h at 1,033 bar.

**
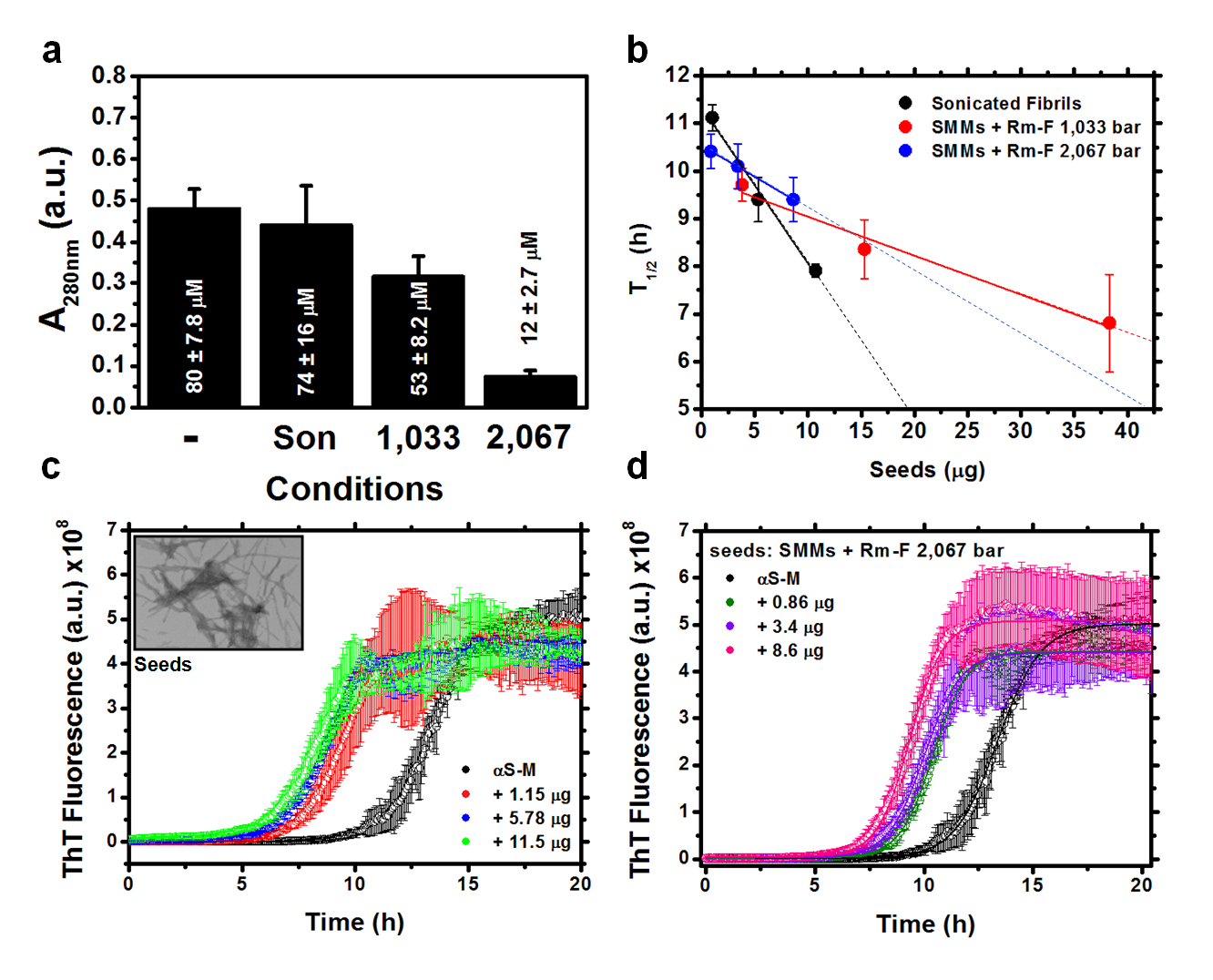
**

**Supplementary Figure 9. Seeding experiments of α-syn species. (a)** Control experiment to estimate the amount of α-syn fibril dissociation for different seeding conditions (i.e., sonication and HHP). Washed fibrils (-) were subjected to sonication (Son) or HHP treatment for 1 h at 1,033 and 2,067 bar. Following these treatments, seeds were harvested at 21,800 g, 5 min, 4 °C, and dried pellets were resuspended in 100 µL of 5 M guanidinium chloride and measured for A_280nm_. Concentrations inside the bars represent the amount of α-syn monomers dissociated from fibrils due to dissolution with 5 M guanidinium chloride after each seeding condition. Experiments are shown as avg. ± s.e.m. (*n* = 4, different fibrillation reactions). **(b)** *t*_1/2_ values obtained at the midpoint of the transitions as a function of the micrograms of different α-syn seeds. Aggregation kinetics of α-syn monomers in the absence of seeds (black spheres) or in the presence of increasing concentrations of **(c)** α-syn fibrils and **(d)** SMMs plus remaining fibrils (Rm-F) formed after 1 h incubation of fibrils at 2,067 bar.

**
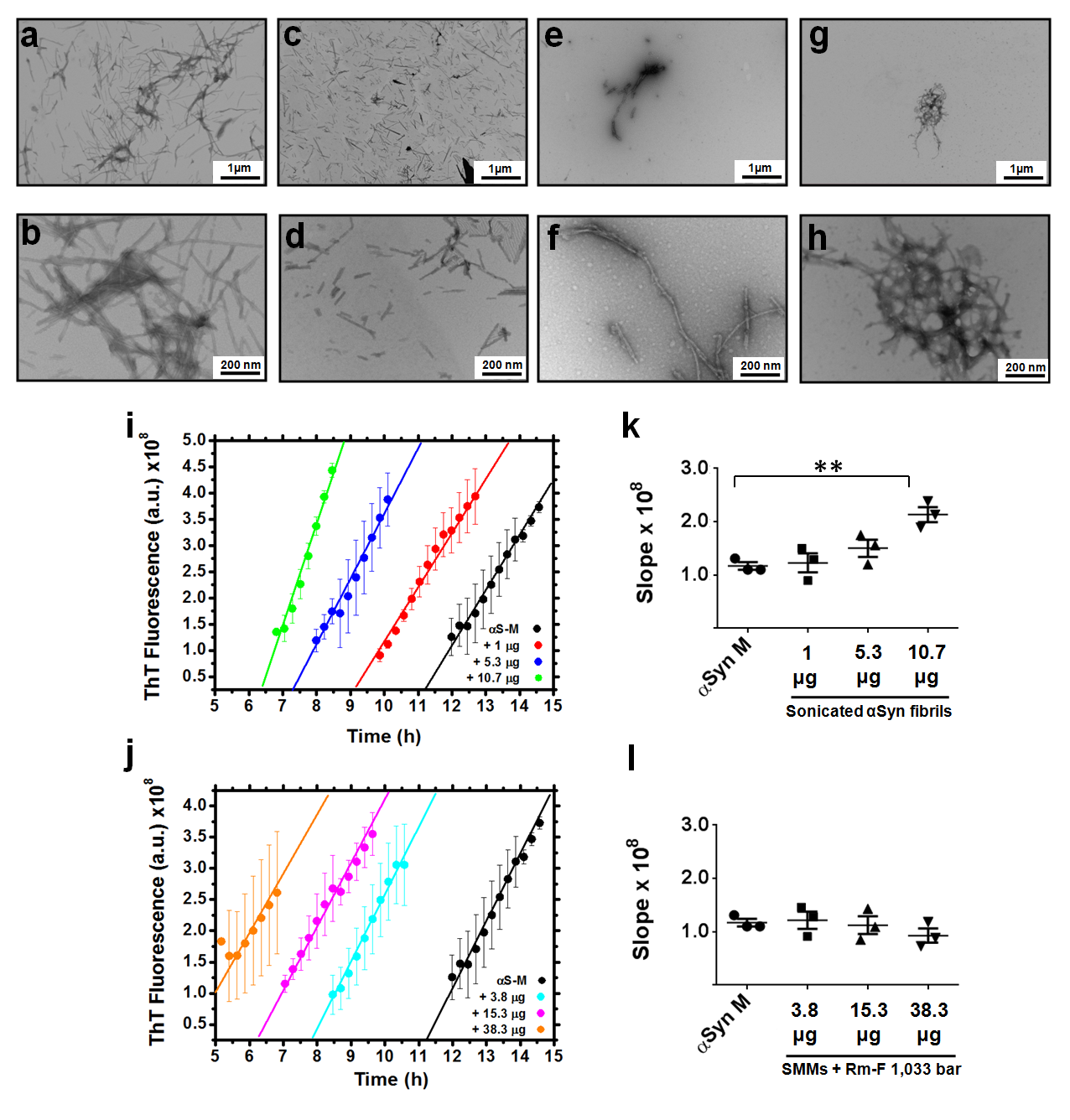
**

**Supplementary Figure 10. Microscopy of seeds and transition analysis. (a-h)** negative staining transmission electron microscopy images of **(a, b)** α-syn fibrils, **(c, d)** pre-formed fibrils generated after sonication, **(e, f)** fibrils after 10 h at 516 bar, and **(g, h)** after 1 h at 1,033 bar. The magnification used was 20,000x for a, c, e and g and 85,000x for b, d, f and h. **(i, j)** Linear fits of the elongation phase for α-syn aggregation experiments using sonicated fibrils and SMMs + Rm-F after 1 h /1,033 bar as seeds, respectively. **(k, l)** Slope values for each curve showing the involvement of secondary events when sonicated fibrils are used as seeds. Statistical analysis was performed using one-way ANOVA, followed by Dunnett`s test for post-hoc comparisons. **, *p* < 0.01. Values represent avg. ± s.e.m. (*n*= 3, independent wells, same kinetic measurement).
